# Precise mimicry of physiological Ca^2+^ oscillations for mammalian oocyte activation by nanosecond pulsed electric field

**DOI:** 10.1002/btm2.70094

**Published:** 2025-12-05

**Authors:** Yi‐Dan Sun, Tong An, Rong Liang, Yu‐Wen Luo, Hong‐Ze Xia, Lei Fu, Shuo Han, Yi‐Xiao Zhu, Zi‐Yi Song, Xue‐Yan Bai, Yao Fu, Xiang‐Wei Fu, Yun‐Peng Hou, Qun Lu

**Affiliations:** ^1^ State Key Laboratory of Animal Biotech Breeding, College of Biological Sciences China Agricultural University Beijing China; ^2^ Medical Center for Human Reproduction, Beijing Chao‐Yang Hospital Capital Medical University Beijing China; ^3^ Department of Obstetrics and Gynecology Peking University People's Hospital Beijing China; ^4^ National Engineering Laboratory for Animal Breeding, College of Animal Science and Technology China Agricultural University Beijing China

**Keywords:** artificial oocyte activation, Ca^2+^ oscillations, infertility treatment, nanosecond pulsed electric field

## Abstract

Oocyte activation deficiency is a primary cause of fertilization failure following intracytoplasmic sperm injection, a problem that can potentially be overcome through artificial oocyte activation (AOA). However, concerns persist regarding the safety and efficacy of AOA in clinical practice. We demonstrated that single‐pulse nanosecond pulsed electric field (nsPEF) stimulation induced Ca^2+^ signaling patterns that depend on intensity in both mouse and human oocytes, facilitating parthenogenetic activation and blastocyst formation. The sperm‐initiated physiological Ca^2+^ oscillations were effectively replicated by a series of Ca^2+^ signals triggered by repeated nsPEF at low or medium intensities, resulting in a significantly higher developmental potential for activated oocytes compared to those treated with A23187 (78.13% vs. 26.70%). The nsPEF stimulation achieved precise manipulation of calcium signaling through two distinct mechanisms: low‐intensity nsPEF pulses mediated repetitive extracellular Ca^2+^ influx in an electro‐permeable manner, while medium‐intensity nsPEF stimulation triggered periodic Ca^2+^ release from the endoplasmic reticulum via the PIP_2_–IP_3_–IP_3_R pathway, generating intracellular Ca^2+^ oscillations that resemble physiological patterns. The non‐invasive nsPEF procedure ensured the safety of oocyte activation by maintaining cellular integrity and minimizing stress responses. The efficacy of nsPEF exposure in precisely manipulating Ca^2+^ signaling patterns is also demonstrated in human mature oocytes. This study establishes a quantitative, non‐invasive nsPEF protocol for AOA that mimics the activation signaling delivered by sperm. This innovative approach overcomes the limitations of conventional chemical activators by enhancing biosafety and clinical efficacy, particularly for patients experiencing total fertilization failure due to severe male infertility. Its ability to accurately regulate Ca^2+^ signaling presents significant potential for advancing research in various fields, including embryonic development and germ cell differentiation.

## Graphical abstract

The figure shows the formation of cytoplasmic Ca^2+^ oscillations induced by nanosecond pulsed electric field (nsPEF). First, nsPEFs stimulation induces extracellular Ca^2+^ and/or Ca^2+^ in endoplasmic reticulum (ER) flow into the cytoplasm through nanopores, then the elevated [Ca^2+^]_i_ is rapidly restored by replenishment of organelles. Under the medium‐intensity stimulation, phosphatidylinositol 4,5‐bisphosphate hydrolysis and inositol 1,4,5 triphosphate generation trigger Ca^2+^ release from ER to form a spontaneous secondary [Ca^2+^]_i_ increase, and its restoration is in dependence on Ca^2+^‐permeable channels and refilling of calcium reservoirs.

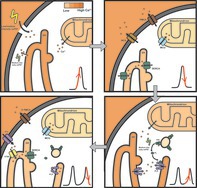

**Translational Impact Statement**

Our findings position nanosecond pulsed electric field (nsPEF) as a superior alternative to pharmacological approaches, combining high safety and efficacy with reduced off‐target effects, thereby advancing the clinical translatability of artificial oocyte activation technologies. Beyond reproductive medicine, the nsPEF's unique capacity for precise spatiotemporal orchestration of calcium signaling dynamics extends its utility, enabling novel applications in developmental engineering through targeted modulation of calcium‐dependent epigenetic reprogramming pathways.

## 1 INTRODUCTION

Infertility affects approximately 17.5% of reproductive‐aged couples globally,^1^ with assisted reproductive technologies (ART) serving as critical interventions. As a cornerstone of ART, in vitro fertilization and embryo transfer (IVF–ET) have successfully addressed a significant proportion of infertility cases. However, intracytoplasmic sperm injection (ICSI) has emerged as the preferred technique in cases of severe male factor infertility or conventional IVF failure.^2^ Despite ICSI implementation, approximately 30% of couples exhibit suboptimal fertilization rates (<30%), while 3–5% experience total fertilization failure (TFF) with a high recurrence risk.^3^ This pattern of recurrent TFF imposes a substantial physical burden and psychological distress on infertile populations.

The primary cause of recurrent TFF following ICSI is often linked to sperm‐related issues, including globozoospermia, teratozoospermia, severe oligozoospermia, or non‐obstructive azoospermia,^4^ which are implicated in varying degrees of oocyte activation deficiency (OAD).^5^, ^6^ These defective sperm fail to fertilize largely by blocking the activation signal in oocytes.^7^, ^8^ Notably, spermatozoa from affected males demonstrate deficient or mutated phospholipase C zeta (PLCζ), impairing physiological intracellular calcium ions ([Ca^2+^]_i_) oscillations at fertilization.^9^, ^10^ As the primary physiological trigger of activation signaling, the PLCζ catalyzes the hydrolysis of phosphatidylinositol 4,5‐bisphosphate (PIP_2_) to inositol 1,4,5 triphosphate (IP_3_), then initiates the release of Ca^2+^ from the endoplasmic reticulum (ER).^11^ The Ca^2+^ elevation subsequently activates calcium‐induced calcium release (CICR) and store‐operated Ca^2+^ entry (SOCE) pathways—dual regulatory mechanisms that govern intracellular Ca^2+^ homeostasis and generate periodic Ca^2+^ oscillatory patterns in oocytes. The spatiotemporal dynamics of Ca^2+^ oscillations constitute biochemical regulators critical for both the initiation of oocyte activation and subsequent embryonic programming.^12^, ^13^

Artificial oocyte activation (AOA) was developed to rescue oocytes that defective sperm fail to activate. By artificially elevating intracellular Ca^2+^ levels, this approach compensates for defective sperms to trigger the resumption of oocyte meiosis.^14^ Three primary AOA methods are currently employed: chemical, mechanical, and electrical methods.^3^ In clinical practice, the chemical activation method, such as calcium ionophore (A23187), is predominantly used.^15^, ^16^, ^17^ The activation rate, embryo quality, clinical pregnancy rate, and live birth rate of ICSI‐AOA cycles are improved compared with the previous conventional ICSI cycle.^18^, ^19^, ^20^ However, current chemical methods primarily elicit monophasic Ca^2+^ transient that deviates from the physiological oscillatory patterns observed in normal fertilization.^14^, ^21^ Additionally, the ICSI‐AOA exhibits marked interstudy variability in activation efficiency (11.4–82.5%) and embryo developmental competence (blastocyst formation rate: 20.9–42.0%) across clinical cohorts,^12^, ^22^, ^23^ highlighting the urgent need for physical activation strategies.

The electrical activation represents a prominent physical AOA method. Conventional protocols employ microsecond‐to‐millisecond electrical pulses to generate micropores in the plasma membrane, enabling extracellular Ca^2+^ influx.^24^, ^25^ Human unfertilized oocytes exposed to electrical stimulation can complete the second meiosis and initiate early embryonic development.^26^ However, longer pulse durations present considerable challenges.^27^ Extended pulse duration increases pore diameter, compromises membrane repair mechanisms, and elevates apoptosis risk^28^, ^29^, ^30^—factors that collectively hinder clinical translation of electrical AOA strategy.

The emergence of nanosecond pulsed electric fields (nsPEF) offers distinct advantages through non‐thermal electroporation mechanisms. These high‐voltage, ultra‐short pulses (1–300 ns) induce simultaneous bioeffects on both plasma membranes and organelle membranes,^31^, ^32^, ^33^ triggering a biphasic Ca^2+^ response.^34^ The Ca^2+^‐rich storage organelles (such as ER and mitochondria) in oocytes also exhibit nsPEF‐induced membrane nanodomain restruction, facilitating coordinated Ca^2+^ mobilization.^35^, ^36^ This transient nanoporation process fundamentally differs from conventional electroporation, as it generates reversible nanoscale pores rather than permanent membrane damage. While calcium signaling is recognized as critical for oocyte activation, current nsPEF protocols lack precise control over Ca^2+^ dynamics to mimic physiological patterns. Therefore, it seems to be urgent to develop quantitatively controllable AOA systems that can induce physiological Ca^2+^ release, while also exploring their mechanistic links to embryonic development and epigenetic reprogramming.

This study aims to establish optimal and safe operational nsPEF parameters to achieve effective oocyte activation while maintaining cellular viability. We systematically investigate the intrinsic mechanisms underlying the generation and maintenance of periodic Ca^2+^ oscillations triggered by nsPEF. Based on nsPEF‐induced various [Ca^2+^]_i_ dynamics in human oocytes, we identify the critical nsPEF's biophysical threshold—including electric field intensity and pulse number—enabling precise control of Ca^2+^ signaling. These findings bridge nsPEF biophysics with reproductive physiology by establishing a controllable Ca^2+^ signaling procedure, ultimately advancing the development of non‐invasive, physiologically mimetic activation protocols for clinical applications.

## 2 RESULTS

### 2.1 Cytoplasmic Ca^2+^ signal patterns are quantitatively manipulated by nsPEF stimulation

**FIGURE 1:**
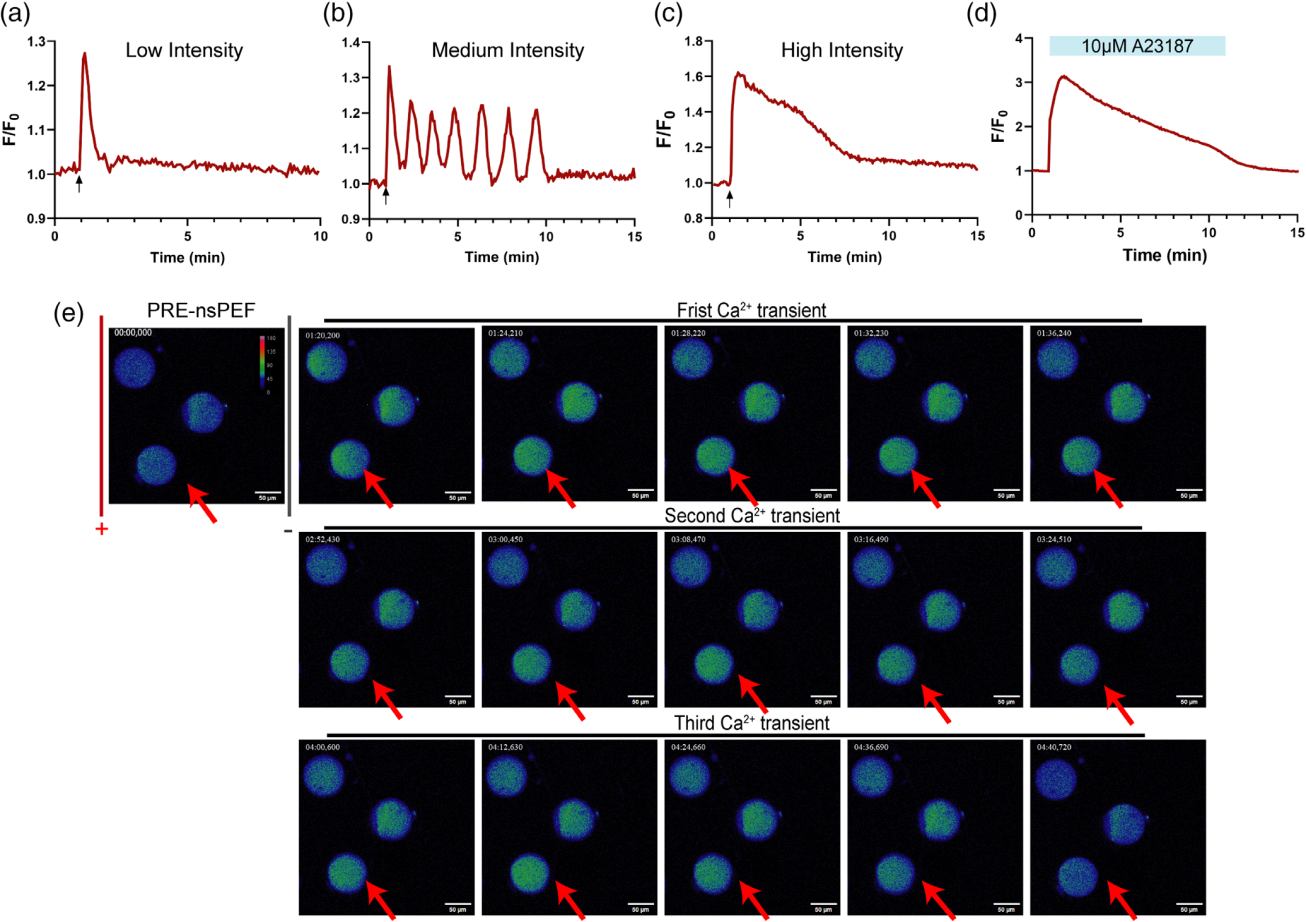
The different patterns of [Ca^2+^]_i_ responses induced by nsPEF stimulation. (a) The pattern of Ca^2+^ response in mouse oocytes stimulated by low‐intensity nsPEF (*n* = 26). (b) The pattern of Ca^2+^ oscillations in oocytes stimulated by medium intensity nsPEF (*n* = 24). (c) The pattern of Ca^2+^ response in oocytes stimulated by high intensity nsPEF (*n* = 17). (d) The patterns of Ca^2+^ response in oocytes treated by 10 μM A23187 (*n* = 17). The red line is the representative response from different oocytes in at least three independent experiments (nsPEF pulses—arrows). (e) Representative images of cytoplasmic Ca^2+^ oscillations after nsPEF exposure at medium intensity. The oocyte indicated by the red arrow exhibited persistent Ca^2+^ oscillations. Scale bar: 50 μm. nsPEF, nanosecond pulsed electric field.

The patterns of [Ca^2+^]_i_ signaling in response to nsPEF with different electric field intensities are represented in Figure 1a–c. After nsPEF stimulation at low intensity, oocytes exhibited a single and rapid [Ca^2+^]_i_ spike (Figure 1a). When the pulse intensity was raised to a moderate level, a specific sustained [Ca^2+^]_i_ oscillation was observed in the ooplasm, consisting of 5–8 Ca^2+^ spikes (Figure 1b), which had nearly the same shape as the spontaneous [Ca^2+^]_i_ oscillations at fertilization. At a relatively high field intensity, the pulse‐induced Ca^2+^ wave displayed a swift [Ca^2+^]_i_ increase, followed by a slow decrease and gradual return to stable [Ca^2+^]_i_ concentration (Figure 1c), which was similar to the [Ca^2+^]_i_ wave induced by chemical activator A23187, as shown in Figure 1d.

Surprisingly, oocytes exhibited the unique spatiotemporal characteristics of [Ca^2+^]_i_ response after the moderate‐intensity nsPEF stimuli (Figure 1e). Upon pulses of nsPEF, the fluorescence intensity in the ooplasm brightened immediately near the anodal side, followed by swift diffusion of the brighter fluorescence throughout the entire oocyte in a few seconds. At this time, [Ca^2+^]_i_ increased rapidly, upregulated by 30.27%, and then returned to its initial state. Subsequently, an unprompted [Ca^2+^]_i_ spike formed at around 1.35 min; meanwhile, the Ca^2+^ level in the whole cytoplasm increased and decreased uniformly. Then, the spontaneous [Ca^2+^]_i_ oscillations recurred in oocytes without additional nsPEF application (Video S1).

The characteristics of the different [Ca^2+^]_i_ responses from each group were set out in Table 1. There was no significant difference in the amplitude of [Ca^2+^]_i_ elevation between the low‐intensity group and the medium‐intensity group (*p* = 0.4611). The duration and the rise rate of one [Ca^2+^]_i_ transient induced by nsPEF were significantly higher as pulse intensity increased, showing a field intensity dependence in the nsPEF‐induced [Ca^2+^]_i_ responses. Moreover, each parameter of [Ca^2+^]_i_ spike induced by A23187 was significantly higher than the counterpart in the high‐intensity group. Collectively, what stands out in Figure 1 is the variability of [Ca^2+^]_i_ response profiles induced by different activation methods.

**TABLE 1:** Indications of [Ca^2+^]_i_ responses induced by nsPEF stimulation and A23187.

Group	*n*	The amplitude of Ca^2+^ elevation (a.u.)^a^	The duration of Ca^2+^ response (min)^a^	The rise rate of Ca^2+^ waves (%)^a^	The decay rate of Ca^2+^ waves (%)
Low intensity	26	1.34 ± 0.02^###^	0.92 ± 0.02^###^	1.22 ± 0.05^###^	0.87 ± 0.03^###^
Medium intensity	24	1.37 ± 0.02^###^	10.81 ± 0.73***	1.80 ± 0.13**,^##^	1.26 ± 0.05**,^###^
High intensity	17	1.60 ± 0.03***,^##^	6.92 ± 0.43***, ^###^	3.33 ± 0.24***	0.13 ± 0.01***
A23187	17	2.42 ± 0.17***	11.10 ± 0.30***	4.19 ± 0.55***	0.19 ± 0.02***

*Note*: Data are mean ± *SEM*. The Shapiro–Wilk normality test was performed for each experimental group. If data from all groups within a comparison passed the normality test (*p* > 0.05), the original one‐way ANOVA was employed; If the indication from any group within a comparison significantly deviated from normality (*p* < 0.05), the corresponding non‐parametric test (Kruskal–Wallis test followed by Dunn's post hoc test) was employed.

Abbreviation: nsPEF, nanosecond pulsed electric field; ANOVA, Analysis of Variance.

^a^ As data from any groups within a comparison significantly deviated from normality, the Kruskal–Wallis test was employed for differential analysis.

*p < 0.05, **p < 0.01, and ***p < 0.001 were considered significantly different compared to the low‐intensity group.

^#^ *p* < 0.05, ^##^ *p* < 0.01, and ^###^ *p* < 0.001 were considered significantly different compared to the A23187 group.

### 2.2 Electrically controlled Ca^2+^ signaling impacts parthenogenetic activation and preimplantation development in mouse oocytes

**FIGURE 2:**
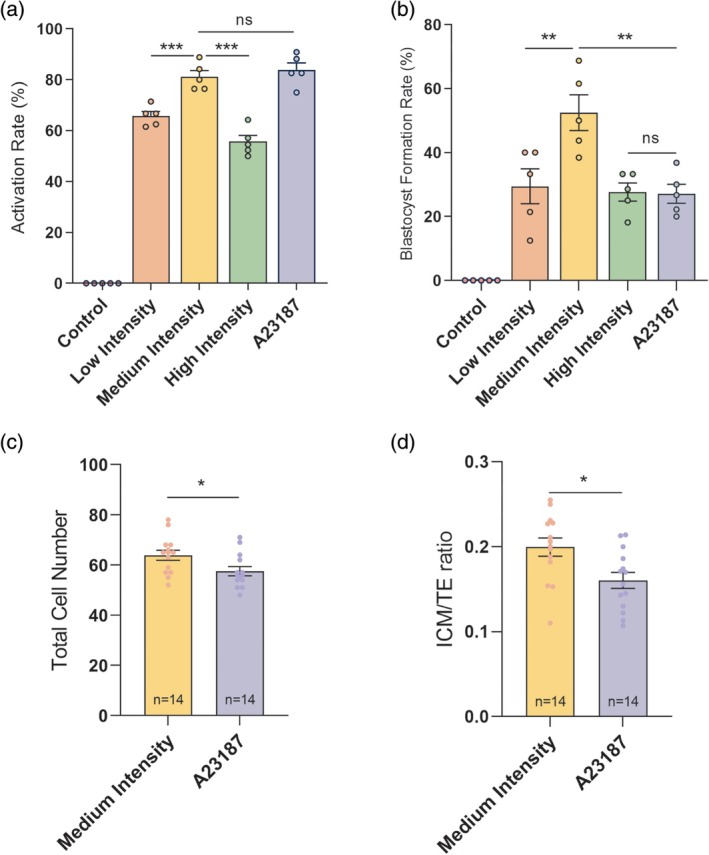
The parthenogenetic activation and developmental outcomes of oocytes after nsPEF stimulation and A23187 treatment. (a) The activation rate of oocytes in different intensity nsPEF treatment groups, A23187 treatment group, and non‐stimulation group (control group), respectively. The activation rate is calculated as the ratio of the number of activated oocytes to the total number of treated oocytes. (b) The blastocyst formation rate of oocytes in different groups. This rate is determined by the ratio of developed blastocysts to the total number of activated oocytes. Error bars represent the mean ± *SEM* of five independent biological replicate experiments (*n* = 5, containing 60, 79, 86, 81, and 84 oocytes in each group, respectively). These data passed the normality test (*p* > 0.05), and the statistical comparison was made with one‐way Analysis of Variance (ANOVA). (c) The total cell number of blastocysts in each treatment group. (d) The ICM/TE ratio of blastocysts in each treatment group. The data are presented as mean ± *SEM*. These data passed the normality test (*p* > 0.05), and the statistical comparison was made with the unpaired t‐test. *p < 0.05, **p < 0.01, ***p < 0.001, ns indicates non‐significant (*p* > 0.05). ICM, inner cell mass; nsPEF, nanosecond pulsed electric field; TE, trophoblast; ANOVA, Analysis of Variance.

We next evaluated whether different pulse‐induced Ca^2+^ signaling patterns activate mouse oocyte development in vitro. Oocytes subjected to nsPEF at each field intensity underwent completion of meiosis and pronuclear (PN) formation. The activation rate in the medium‐intensity group was significantly higher than in the other intensity groups of nsPEF stimulation, but not significantly different from that in the A23187 group (81.21% vs. 87.27%, *p* = 0.8974, Figure 2a).

The parthenogenetic‐activated oocytes from each group further developed to the blastocyst stage, and there were obvious differences in blastocyst formation rates. The blastocyst formation rate in the medium‐intensity nsPEF group was the highest, which was significantly higher than that in the A23187 group (51.67% vs. 26.70%, *p* = 0.0016) (Figure 2b), suggesting that medium‐intensity nsPEF improves the developmental potential of activated oocytes compared to A23187 treatment.

Additionally, the developmental quality of parthenogenetic blastocysts was assessed between the medium‐intensity nsPEF group and the A23187 group. The parthenogenetic blastocysts in the medium‐intensity nsPEF group had a higher total cell number (62.43 vs. 57.5, *p* = 0.0258) and a higher inner cell mass (ICM)/trophoblast (TE) ratio (0.20 vs. 0.16, *p* = 0.0275) compared to those in the A23187 group (Figure 2c,d), indicating superior embryo quality in the medium‐intensity nsPEF group.

### 2.3 Fertilization‐like Ca^2+^ oscillations simulated by multiple pulses increase oocyte activation efficiency and developmental potentials

**FIGURE 3:**
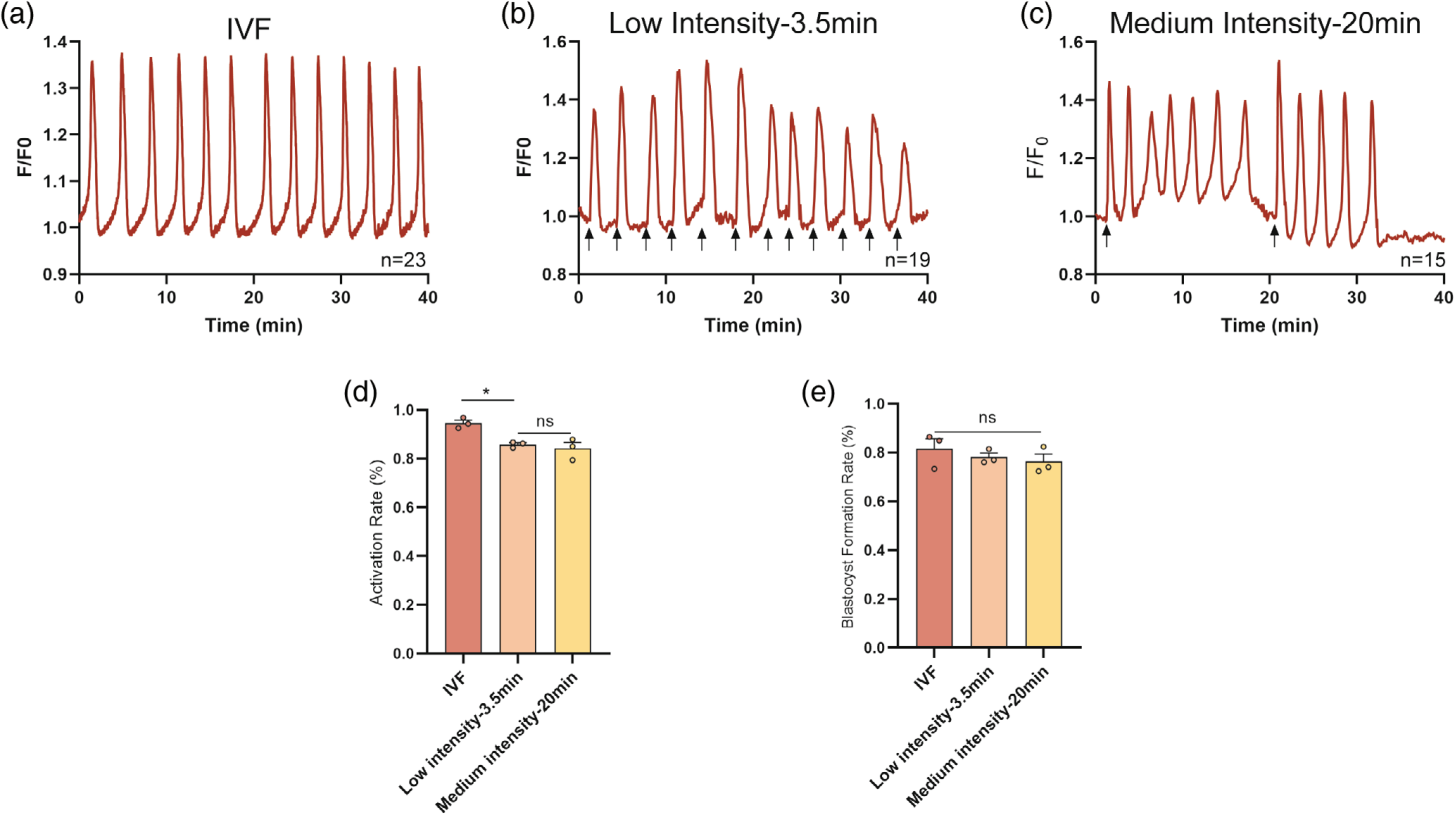
The effect of fertilization‐like Ca^2+^ oscillations induced by repetitive nsPEF stimulation on oocyte activation and development. (a–c) The different patterns of [Ca^2+^]_i_ oscillations induced by nsPEF stimulation and IVF in mouse oocytes. (A) The pattern of Ca^2+^ oscillations in oocytes during IVF. (b) The pattern of Ca^2+^ response stimulated by low intensity nsPEF at 3.5 min intervals. (c) The pattern of Ca^2+^ response stimulated by medium intensity nsPEF at 20‐min intervals (nsPEF pulses—arrows). (d) The activation rates of oocytes in different intensity nsPEF treatment groups and the IVF group (control group), respectively. (e) The blastocyst formation rates of oocytes in different groups. Error bars represent the mean ± *SEM* of three independent biological replicate experiments (*n* = 3, containing 106, 91, and 107 oocytes in each group, respectively). These data passed the normality test (*p* > 0.05), and the statistical comparison was made with one‐way ANOVA (Analysis of Variance). *p < 0.05, ns indicates non‐significant (*p* > 0.05). IVF, in vitro fertilization; nsPEF, nanosecond pulsed electric field.

The prominent physiological Ca^2+^ changes observed after fertilization are long‐lasting and repetitive increases until PN formation. The prolonged time duration of nsPEF‐induced Ca^2+^ signaling is probably beneficial for oocyte activation and development in vitro; thus, the sperm‐induced oscillatory Ca^2+^ signals were reproduced by repetitive nsPEF pulses in mouse oocytes. According to the characteristics of physiological Ca^2+^ oscillation detected during IVF (Figure 3a and Table 2), oocytes were repetitively exposed to low‐intensity pulses every 3.5 min or medium‐intensity pulses every 20 min (applied after spontaneous Ca^2+^ signal termination) for 2 h to reproduce the physiological Ca^2+^ oscillation required for oocyte activation (Figure 3b,c). The comparison of the characteristics of Ca^2+^ oscillation in each group revealed that the [Ca^2+^]_i_ transients elicited by the nsPEF pulses, at either low or moderate intensity, typically exhibit steeper rates of elevation and recovery than the natural [Ca^2+^]_i_ spikes (Table 2). In contrast, the spontaneous sustained [Ca^2+^]_i_ oscillations in the oocyte after the moderate‐intensity nsPEF stimulation more closely resemble the pattern of physiologic [Ca^2+^]_i_ oscillation in the IVF group (*p* = 0.307 and *p* = 0.260), even though the Ca^2+^ spikes occur at shorter intervals in the medium‐intensity group (*p* < 0.001). Further, compared to single nsPEF stimulation, oocytes exhibited higher parthenogenetic activation efficiency (85.75% and 84.10%, Figure 3d) and blastocyst formation rate (78.13% and 76.28%, Figure 3e) under the electro‐induced physiological‐like [Ca^2+^]_i_ oscillations. Whereas distinctions in [Ca^2+^]_i_ signaling induced by different nsPEF stimuli did not lead to differences in oocyte activation and development (*p* = 0.769 and *p* = 0.909), this implies that the Ca^2+^ signaling required for activation may be in a cumulative form instead of strictly dependent on the specific characteristics of the Ca^2+^ spikes.

**TABLE 2:** Indications of [Ca^2+^]_i_ responses in different treatment groups.

Group	*n*	The amplitude of Ca^2+^ elevation (a.u.)^a^	The rise rate of Ca^2+^ spikes (%)^a^	The decay rate of Ca^2+^ spikes (%)	The interval of Ca^2+^ spikes (s)^a^
Low intensity	19	1.34 ± 0.01	1.19 ± 0.05^##^	0.89 ± 0.03^###^	205.18 ± 6.60
Medium intensity—first Ca^2+^ spike	15	1.38 ± 0.02	1.95 ± 0.16*, ^###^	1.26 ± 0.06***,^#^	74.26 ± 5.16***, ^###^
Medium intensity—spontaneous Ca^2+^ spikes	35	1.31 ± 0.03	0.98 ± 0.06*	0.98 ± 0.03	
IVF	23	1.36 ± 0.01	0.88 ± 0.04**	1.05 ± 0.02***	214.60 ± 8.59

*Note*: Data are mean ± *SEM*. The Shapiro–Wilk normality test was performed for each experimental group. If data from all groups within a comparison passed the normality test (*p* > 0.05), the one‐way ANOVA was employed; If the indication from any group within a comparison significantly deviated from normality (*p* < 0.05), the corresponding non‐parametric test (Kruskal–Wallis test followed by Dunn's post hoc test) was employed.

^a^ As data from any groups within a comparison significantly deviated from normality, the Kruskal–Wallis test was employed for differential analysis.

*p < 0.05, **p < 0.01, and ***p < 0.001 were considered significantly different compared to the low‐intensity group.

^#^ *p* < 0.05, ^##^ *p* < 0.01, and ^###^ *p* < 0.001 were considered significantly different compared to the IVF group.

### 2.4 Extracellular Ca^2+^ and intracellular Ca^2+^ storage participate in the formation of [Ca^2+^]_i_ oscillation under nsPEF stimulation

**FIGURE 4:**
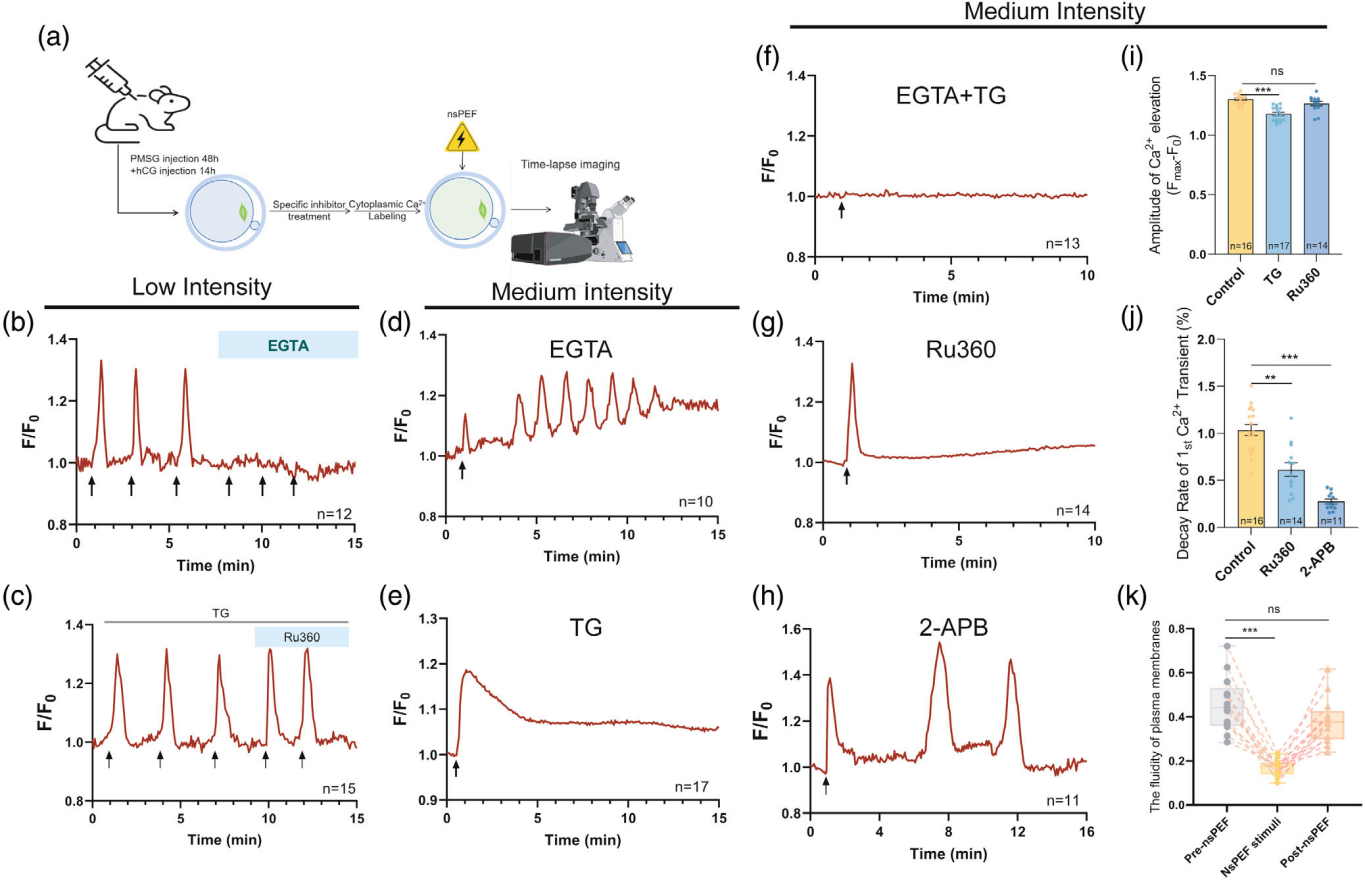
The formation of [Ca^2+^]_i_ oscillations in oocytes upon nsPEF stimulation at low or medium intensity. (a) Schematic showing the procedures for the formation pathway of nsPEF‐induced [Ca^2+^]_i_ responses by antagonist experiment in mouse MII oocytes. (b) The representative [Ca^2+^]_i_ responses to low‐intensity nsPEF stimulation (red line) in oocytes incubated with 5 mM EGTA. (c) The representative [Ca^2+^]_i_ responses to low‐intensity nsPEF stimulation (red line) in oocytes incubated with 10 μM TG and/or 10 μM Ru360. (d) The representative [Ca^2+^]_i_ responses to medium‐intensity nsPEF stimulation (red line) in oocytes incubated with 5 mM EGTA. (e) The representative [Ca^2+^]_i_ responses to medium‐intensity nsPEF stimulation (red line) in oocytes incubated with 10 μM TG (f) The representative [Ca^2+^]_i_ responses to medium‐intensity nsPEF stimulation (red line) in oocytes incubated with both 5 mM EGTA and 10 μM TG. (g) The representative [Ca^2+^]_i_ responses to medium‐intensity nsPEF stimulation (red line) in oocytes incubated with 10 μM Ru360. (h) The representative [Ca^2+^]_i_ responses to medium‐intensity nsPEF stimulation (red line) in oocytes incubated with 10 μM 2‐APB (nsPEF pulses—arrows). (i) The amplitude of the first [Ca^2+^]_i_ transient induced by medium‐intensity nsPEF from different treatment groups. (j) The decay rate of the first [Ca^2+^]_i_ transient induced by medium‐intensity nsPEF from different treatment groups. (k) The fluidity of plasma membrane on oocytes was detected before stimulation, upon stimulation and after medium‐intensity nsPEF stimulation. The data are presented as mean ± *SEM*. These data passed the normality test (*p* > 0.05), and the statistical comparison was made with one‐way ANOVA. **p < 0.01, ***p < 0.001, ns indicates nonsignificant (*p* > 0.05). EGTA, Ethylene Glycol Tetraacetic Acid; 2‐APB, 2‐aminoethyl diphenylborinate; MII, metaphase II; nsPEF, nanosecond pulsed electric field; TG, thapsigargin; ANOVA, Analysis of Variance.

To clarify the formation mechanism of the fertilization‐like [Ca^2+^]_i_ oscillations elicited by nsPEF pulses, we took advantage of inhibitors to selectively remove free Ca^2+^ from specific regions (Figure 4a). First, Ca^2+^ spikes were completely blocked under 0 mM extracellular Ca^2+^ conditions in oocytes treated with EGTA (Ethylene Glycol Tetraacetic Acid), after the low‐intensity nsPEF stimulation (Figure 4b), with the exception that removal of calcium storage from the ER and mitochondria using TG and Ru360 did not compromise the nsPEF‐induced Ca^2+^ elevation (Figure 4c). However, it is apparent from Figure 4d that sustained [Ca^2+^]_i_ oscillations could still develop without extracellular Ca^2+^ supplementation after the medium‐intensity nsPEF stimulation. These oscillatory Ca^2+^ signaling exhibited a lower amplitude (*p* = 0.0003), a prolonged duration (*p* = 0.0019), and a protracted interval between [Ca^2+^]_i_ transients than those in untreated oocytes (*p* < 0.0001, Table 3). Therefore, extracellular Ca^2+^ influx is an important component of [Ca^2+^]_i_ oscillation formation induced by nsPEF.

**TABLE 3:** Indications of persistent [Ca^2+^]_i_ oscillations induced by nsPEF in the medium‐intensity group.

Group	*n*	The amplitude of Ca^2+^ elevation (a.u.)	The duration of intact Ca^2+^ oscillations (min)^a^	The interval of first and second Ca^2+^ transient (s)^a^	The number of Ca^2+^ transients^a^
Control	16	1.33 ± 0.01	7.68 ± 0.47	1.45 ± 0.10	5.94 ± 0.41
EGTA	10	1.13 ± 0.01***	10.99 ± 0.47**	3.30 ± 0.11**	5.80 ± 0.57
2‐APB	11	1.23 ± 0.05	11.09 ± 0.78**	6.59 ± 0.85***	3.00 ± 0.17***

*Note*: Data are mean ± *SEM*. The Shapiro–Wilk normality test was performed for each experimental group. If data from all groups within a comparison passed the normality test (*p* > 0.05), the original one‐way ANOVA was employed; If the indication from any group within a comparison significantly deviated from normality (*p* < 0.05), the corresponding non‐parametric test (Kruskal–Wallis test followed by Dunn's post hoc test) was employed.

Abbreviations: EGTA, Ethylene Glycol Tetraacetic Acid; 2‐APB, 2‐aminoethyl diphenylborinate; nsPEF, nanosecond pulsed electric field; ANOVA, Analysis of variance.

^a^ As data from any groups within a comparison significantly deviated from normality, the Kruskal–Wallis test was employed for differential analysis.

*p < 0.05, **p < 0.01, and ***p < 0.001 were considered significantly different compared to the control group.

In the absence of Ca^2+^ from the ER storage, oocytes subjected to medium intensity pulses exhibited a single [Ca^2+^]_i_ wave (Figure 4e) with markedly reduced amplitude (*p* < 0.0001, Figure 4i), instead of repeated [Ca^2+^]_i_ oscillations. When EGTA and TG were simultaneously employed to clear both extracellular Ca^2+^ and ER stores, there was an invisible [Ca^2+^]_i_ alteration after the medium intensity nsPEF stimulation (Figure 4f). This suggests that the extracellular milieu and ER stores are the primary contributors to the nsPEF‐induced first [Ca^2+^]_i_ transient.

We found that synchronized Ca^2+^ oscillations in the cytoplasm and mitochondria were triggered by medium‐intensity nsPEF exposure (Figure S1A,B). To verify that mitochondria stores likewise contribute to the nsPEF‐induced [Ca^2+^]_i_ oscillations, we specifically depleted Ca^2+^ in the mitochondria using 10 μM Ru360 (Figure S1C). In this situation, a single cytoplasmic Ca^2+^ transient was observed in oocytes after nsPEF stimulation (Figure 4g), accompanied by an identical pattern of [Ca^2+^]_mito_ changes (Figure S1D). While the amplitude of [Ca^2+^]_i_ elevation was not significantly altered (Figure 4i, *p* = 0.2064), its decay rate was considerably reduced (Figure 4j, *p* = 0.0025) in oocytes without mitochondria stores upon nsPEF stimulation. This suggests that mitochondria, as the intracellular fast‐response calcium pools, uptake increased [Ca^2+^]_i_ rather than release Ca^2+^ to regulate intracellular calcium homeostasis during the nsPEF‐induced [Ca^2+^]_i_ oscillations.

SOCE, as a refilling mechanism for depleted Ca^2+^ stores in ERs, was inhibited by 10 μM 2‐APB to determine the role of cytosolic Ca^2+^ backflow into the ER after nsPEF stimulation at medium intensity. The nsPEF‐induced [Ca^2+^]_i_ oscillations were significantly attenuated in oocytes (Figure 4h), as evidenced by the prolonged duration of oscillations (*p* = 0.0010), the fewer [Ca^2+^]_i_ transients (*p* < 0.0001), and the longer intervals between [Ca^2+^]_i_ transients (*p* = 0.0008, Table 3). The amplitude of the first Ca^2+^ spiking was comparable (*p* = 0.053, Table 3), but its recovery rate of cytoplasmic Ca^2+^ was significantly lower than that in the untreated group (*p* < 0.0001, Figure 4j), demonstrating that the recovery from Ca^2+^ elevation was compromised due to the lack of SOCE function. The results indicated that the re‐entry of Ca^2+^ into oocytes induced by SOCE was the key point for the formation of periodic [Ca^2+^]_i_ oscillation upon medium‐intensity pulses.

We further investigated whether modifications to the plasma membrane structure were reversible in oocytes stimulated by nsPEF. The plasma membrane conformation of oocytes was highly responsive to nsPEF stimulation (0.44 vs. 0.17, *p* < 0.0001), and this change in membrane fluidity could be restored within a few minutes (0.44 vs. 0.37, *p* = 0.1201), as shown in Figure 4k. This demonstrates that the lifetime of nanopores in the plasma membrane evoked by nsPEF stimuli is limited to only a few minutes, allowing for extracellular Ca^2+^ influx, and then the conformation of the plasma membrane rapidly resumes its original state to prevent excessive extracellular Ca^2+^ uptake.

### 2.5 Maintenance of [Ca^2+^]_i_ oscillations is dependent on IP_3_R activation by two regulatory mechanisms

**FIGURE 5:**
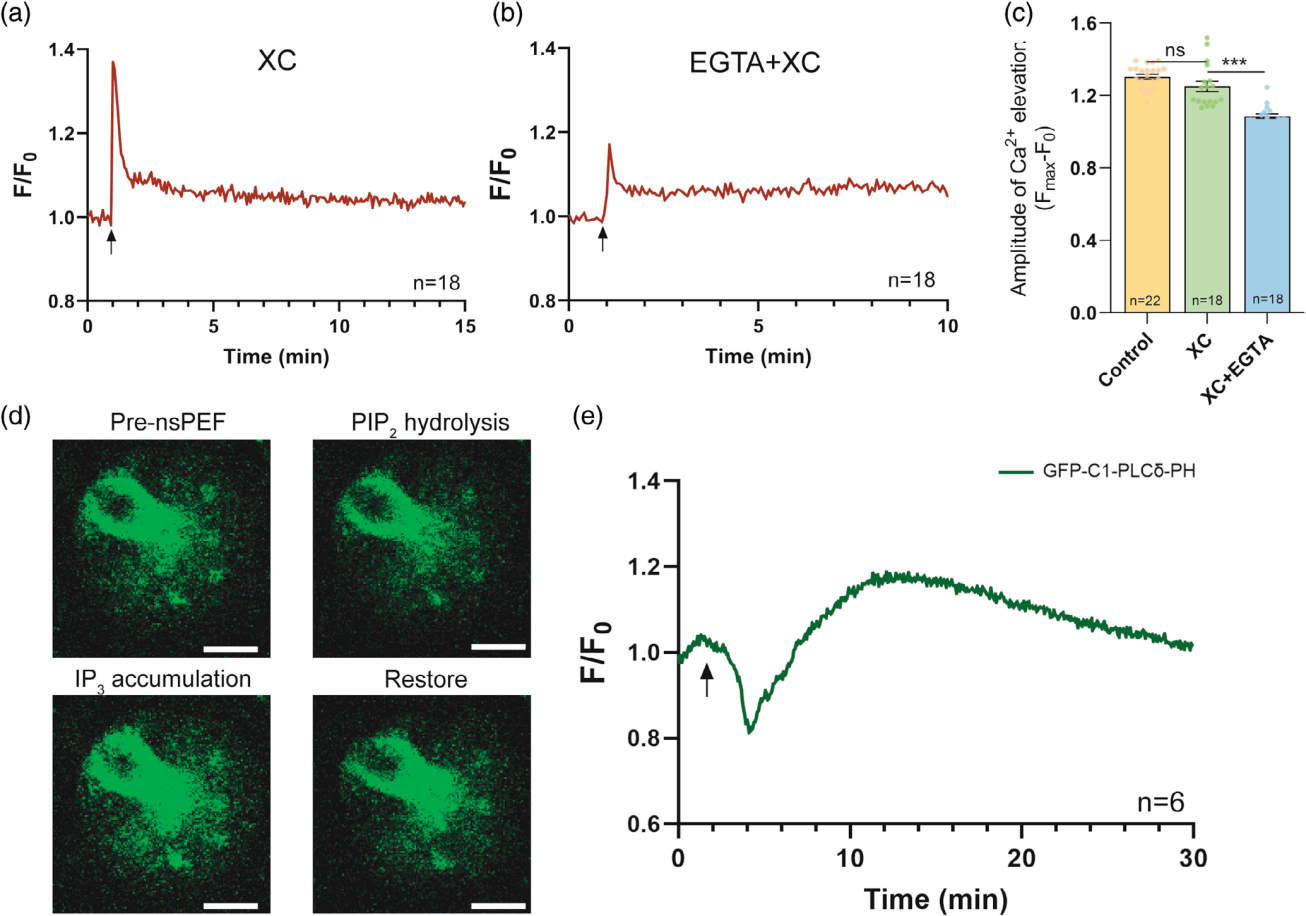
The maintenance mechanism of Ca^2+^ oscillations stimulated by medium‐intensity nsPEF. (a) The representative [Ca^2+^]_i_ responses to medium‐intensity nsPEF stimulation (red line) in oocytes incubated with 10 μM XC (b) The representative [Ca^2+^]_i_ responses to medium‐intensity nsPEF stimulation (red line) in oocytes incubated with both 5 mM EGTA and 10 μM XC. (c) The amplitude of the 1st [Ca^2+^]_i_ transient of [Ca^2+^]_i_ oscillations from different treatment groups. These data significantly deviated from normality (*p* < 0.05), and the statistical comparison was made with the Kruskal–Wallis test. (d) Representative images of cytoplasmic GFP‐C1‐PLCδ‐PH signal alteration. Scale bar: 20 μm. (e) The representative response of GFP‐C1‐PLCδ‐PH fluorescence signal after medium‐intensity nsPEF stimulation (green line) in oocytes (nsPEF pulses—arrows). The data are presented as mean ± *SEM*. ***p < 0.001, ns indicates nonsignificant (*p* > 0.05). nsPEF, nanosecond pulsed electric field; XC, xestospongin C.

Whether the first [Ca^2+^]_i_ elevation induced by the moderate‐intensity nsPEF stimulation reaches the activation threshold of the IP_3_R and CICR occurrence to mediate further Ca^2+^ release from the ERs was explored. When IP_3_R activity was inhibited by Xestospongin C (XC), the [Ca^2+^]_i_ oscillation was prematurely halted in oocytes stimulated by medium‐intensity nsPEF (Figure 5a), indicating that IP_3_R is essential for maintaining nsPEF‐induced [Ca^2+^]_i_ oscillations. There was no significant difference in the first peak of [Ca^2+^]_i_ increase upon nsPEF stimulation between the XC‐treated oocytes and the untreated oocytes, but the oocytes lacking IP_3_R activity exhibited a significantly reduced Ca^2+^ peak in 0 mM extracellular Ca^2+^ milieu (Figure 5b,c). This suggests that nsPEF pulses did not directly trigger Ca^2+^ efflux across IP_3_R channels on ERs. The Ca^2+^ elevation evoked by nsPEF secondarily activates IP_3_R to form spontaneous [Ca^2+^]_i_ oscillations via the CICR pathway.

Based on the model of quantitative regulation of intracytoplasmic Ca^2+^‐IP_3_ kinetics by nsPEF,^36^ we speculate that the generation of IP_3_ similarly activated IP_3_R to maintain [Ca^2+^]_i_ oscillations after nsPEF stimulation. Pre‐exposure GFP‐C1‐PLCδ‐PH fluorescence in oocytes illustrated the presence of intracellular PIP_2_ in vesicular structures (Figure 5d). The green fluorescence decreased rapidly at 2.71 ± 0.30 min after nsPEF exposure, then began to gradually recover at 3.44 ± 0.27 min (Figure 5d,e), implying that nsPEF stimulation promotes intracellular PIP_2_ hydrolysis and increases IP_3_ concentration in oocytes. Under the dual regulation of Ca^2+^ level and IP_3_, the activated IP_3_R mediates Ca^2+^ release from ERs to generate sustained [Ca^2+^]_i_ oscillations.

### 2.6 The application of nsPEF stimulation is a safe method for oocyte activation

**FIGURE 6:**
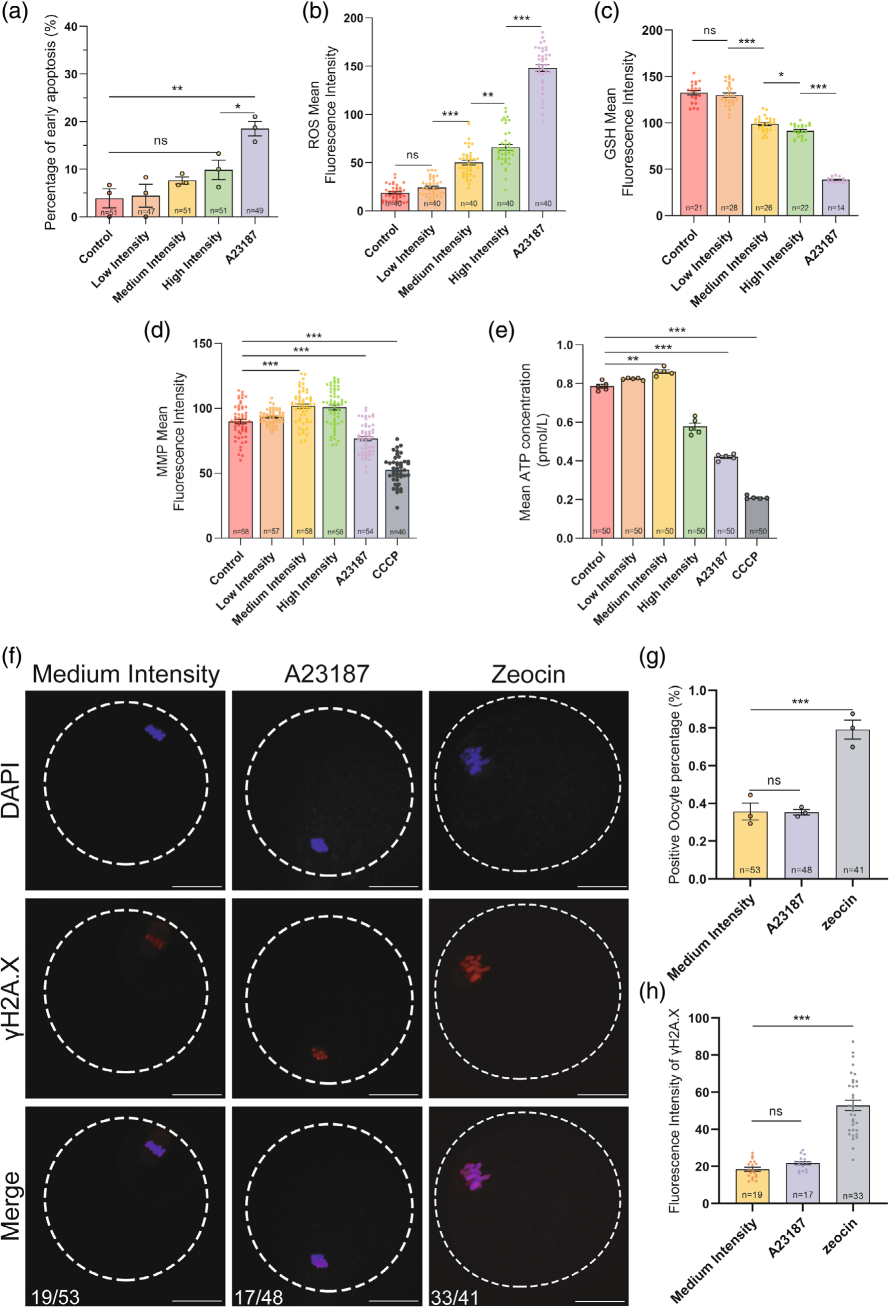
The effects of nsPEF stimulation on cytoplasmic and nuclear security in mouse oocytes. (a) Percentage of early apoptosis of oocytes in each treatment group. (b) Mean fluorescence intensity of intracellular ROS in each treatment group. (c) Mean fluorescence intensity of intracellular GSH in each treatment group. (d) Mean fluorescence intensity of MMP in oocytes in each treatment group. These data passed the normality test (*p* > 0.05), and the statistical comparison was made with one‐way ANOVA. (e) Intracellular ATP content of each oocyte in each treatment group. (f) Representative immunostaining images of oocytes with DNA double‐strand breaks in each treatment group. Scale bar: 20 μm. (g) The percentage of oocytes with DNA damage in each treatment group. (h) The fluorescence intensity of oocytes with DNA damage in each treatment group. The data are presented as means ± *SEM*s. *p < 0.05, **p < 0.01, ***p < 0.001, ns indicates nonsignificant (*p* > 0.05). These data passed the normality test (*p* > 0.05), and the statistical comparison was made with one‐way ANOVA. The exclusion criterion applied to data points was the removal of statistical outliers, which was determined using Tukey's method (values lying beyond 1.5 times the interquartile range). The final *n* value for each experimental group reflects the number of data points remaining after this step. GSH, glutathione; MMP, mitochondrial membrane potential; nsPEF, nanosecond pulsed electric field; ROS, reactive oxygen species; XC, xestospongin C; ANOVA, Analysis of Variance.

The safety of nsPEF applications on mouse oocytes was further examined. The early apoptosis rates of oocytes in the low‐ to high‐intensity nsPEF groups were not significantly different from those in the control (non‐stimulation) group; despite the apoptosis rate in the A23187 groups being highest, all rates remained below 20% (Figure 6a). Detection of intracellular reactive oxygen species (ROS) and glutathione (GSH) concentrations in oocytes from each group revealed greater ROS accumulation and GSH depletion with increasing field intensity of the nsPEF exposure. However, the level of oxidative stress was significantly higher in A23187‐treated oocytes than that in the high‐intensity group (Figure 6b,c).

To investigate whether mitochondria were involved in the nsPEF‐induced oxidative stress response, mitochondrial membrane potential (MMP) and ATP levels were measured in oocytes following treatment with different nsPEF intensities, A23187, or the oxidative phosphorylation uncoupler carbonyl cyanide m‐chlorophenyl hydrazine (CCCP, as a positive control). Notably, significantly higher MMP levels (*p* = 0.0023) and greater ATP concentration (*p* = 0.0341) were observed in oocytes from the medium‐intensity group compared to the control group (Figure 6d,e), suggesting that oocytes stimulated by medium‐intensity nsPEF had higher mitochondrial activity. Conversely, A23187 treatment caused lower MMP (*p* = 0.0049) and reduced ATP content in oocytes (*p* < 0.0001).

The potential impairment of DNA structure across the nuclear envelope by nsPEF pulses was investigated using γH2A.X antibody labeling (Figure 6f). Both nsPEF exposure and A23187 incubation resulted in a lower incidence of abnormal chromosome distribution and DNA double‐strand breaks (Figure 6g) and a lower degree of DNA damage (Figure 6h) compared to zeocin‐treated oocytes (positive control). This suggests that nsPEF stimulation in this protocol is non‐damaging to DNA in oocytes.

### 2.7 Human oocytes exhibit various degrees of Ca^2+^ waves after different parameters of nsPEF stimulation

**FIGURE 7:**
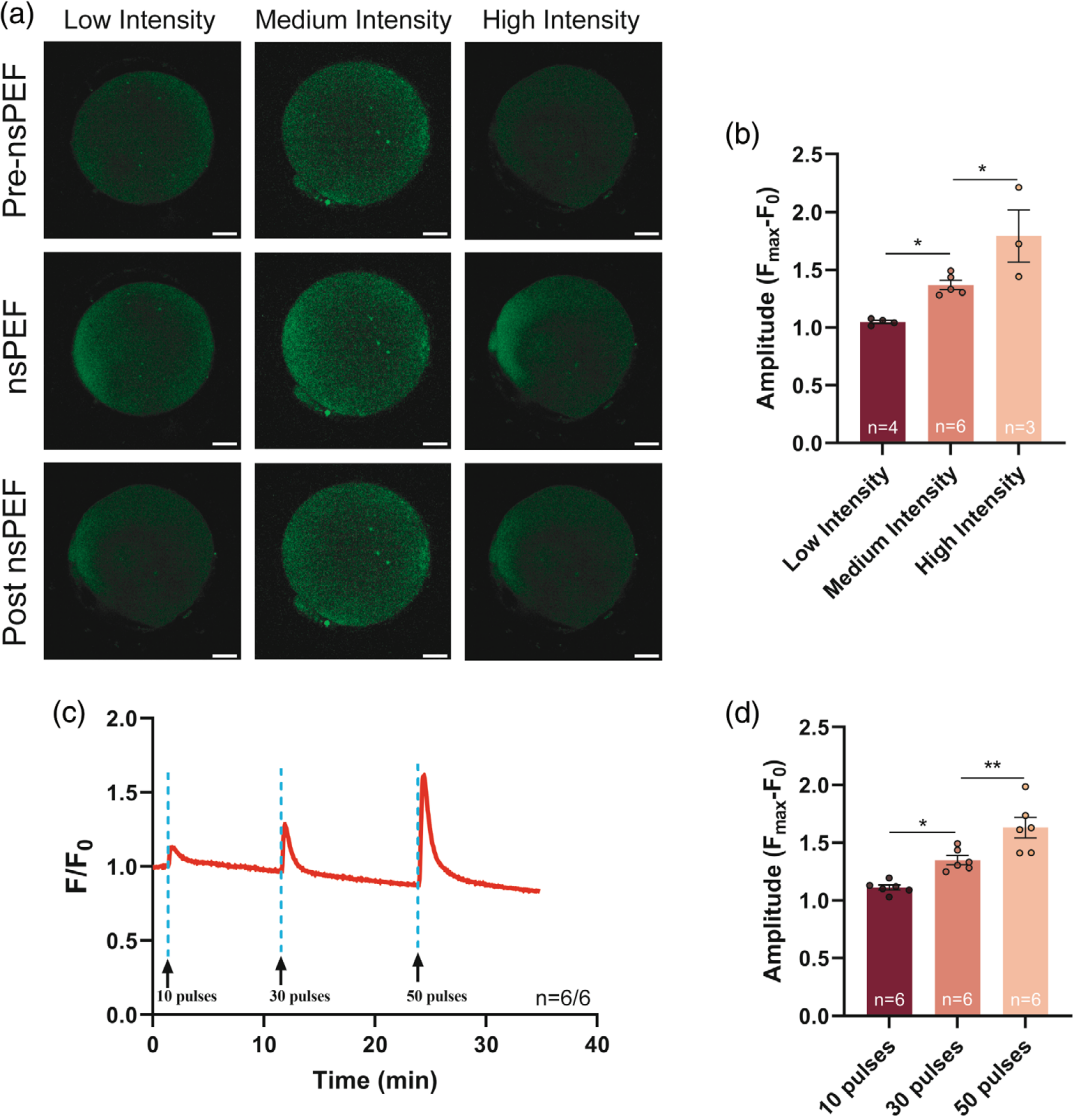
The patterns of cytoplasmic Ca^2+^ waves in human oocytes induced by nsPEF stimulation. (a) Representative images of cytoplasmic Ca^2+^ alteration before and after nsPEF exposure of 10 pulses at low, medium, or high intensity. Scale bar: 20 μm. (b) The amplitude of Ca^2+^ transient induced by nsPEF with different intensities. (c) Representative Ca^2+^ oscillation patterns following repeated nsPEF exposure with medium intensity. (d) The amplitude of Ca^2+^ transient induced by nsPEF with different numbers of pulses. The data are presented as mean ± *SEM*. These data passed the normality test (*p* > 0.05), and the statistical comparison was made with one‐way ANOVA. *p < 0.05, **p < 0.01. nsPEF, nanosecond pulsed electric field; ANOVA, Analysis of Variance.

To investigate how [Ca^2+^]_i_ changes in human oocytes under nsPEF exposure, we found that a single Ca^2+^ transient was induced in the ooplasm when different intensities of nsPEF were applied to the human in vitro matured (IVM)–MII oocytes. Oocytes exhibited a single, minimally visible [Ca^2+^]_i_ wave when stimulated by 10 pulses of low‐intensity nsPEF, while the amplitude of the [Ca^2+^]_i_ wave increased after the medium and high‐intensity stimulation (Figure 7a,b). Oocytes also formed increasing [Ca^2+^]_i_ waves under cumulative pulses of medium‐intensity nsPEF (Figure 7c,d). Cytoplasmic Ca^2+^ concentration was urgently upregulated by 34.84% under 30 pulses of medium‐intensity nsPEF. Thus, varying degrees of Ca^2+^ peaks could be induced by adjusting the intensity or number of nsPEF pulses.

## 3 DISCUSSION

Patients with OAD experience both physical and psychological impairments during repeated ICSI cycles, a challenge that persists despite existing AOA protocols owing to unclear activation mechanisms.^14^ These limitations highlight the urgent need to develop novel AOA strategies with improved efficacy. We propose an electrical AOA method employing nsPEF, specifically designed to preserve oocyte viability while potentiating activation competence and embryonic development by generating physiological‐like sustained Ca^2+^ oscillations. The low‐intensity nsPEF stimulation triggers transmembrane Ca^2+^ influx, whereas the moderate‐intensity stimulation induces synchronized extracellular Ca^2+^ entry coupled with mobilization of intracellular calcium stores, followed by PIP_2_ hydrolysis mediated by nsPEF‐induced Ca^2+^ elevation and subsequent IP_3_R‐dependent calcium homeostasis modulation. This mechanism enables the establishment of physiological‐like Ca^2+^ oscillations in oocytes through multiple nsPEF exposures. In contrast to conventional chemical methods, this physics‐driven strategy eliminates chemical damage while enhancing the developmental potential of activated oocytes. These findings establish nsPEF‐based AOA as a technique that can be quantitatively controlled to modulate calcium signaling, offering the necessary Ca^2+^ dynamics for oocyte activation and embryonic development.

Most AOA methods can only induce a single or a few Ca^2+^ transients in the ooplasm, leading to suboptimal activation and poor embryonic developmental competence.^37^ For instance, although treatments such as A2318, ionomycin, or ethanol elicit a strong Ca^2+^ rise, the resulting activation efficiency is inconsistent, with few early embryos reaching transfer suitability.^14^ Our findings highlight the distinct advantage of the sustained Ca^2+^ oscillations induced by nsPEF as a superior activation strategy compared to traditional chemical methods (e.g., A23187, by far the most popular clinical agents). By adjusting the parameters of nsPEF, we achieved precise control over intracellular Ca^2+^ dynamics, revealing a critical relationship between Ca^2+^ oscillation patterns and activation outcomes. The moderate‐intensity nsPEF triggered sustained Ca^2+^ oscillations characterized by 5 to 8 peaks over approximately 10 min, synchronized with mitochondrial Ca^2+^ dynamics. While achieving activation rates nearly equivalent to those obtained with A23187, this treatment also conferred a significant advantage by improving the blastocyst formation rate in murine oocytes. In contrast, both low‐intensity nsPEF (transient single peak) and high‐intensity nsPEF (non‐oscillatory Ca^2+^ wave resembling A23187's signal) caused suboptimal activation outcomes. These findings align with previous observations indicating non‐physiological Ca^2+^ signals can disrupt ooplasmic homeostasis and hinder preimplantation development.^38^ Mechanistically, the sustained Ca^2+^ oscillations more accurately mimic the activation signaling necessary for fertilization, which potentially facilitates complete cortical granule exocytosis and ensures orderly meiotic progression.^39^, ^40^ Importantly, embryos activated through oscillatory signaling exhibited enhanced morphological grades and blastocyst quality compared to those treated with A23187, underscoring the long‐term developmental benefits of sustained Ca^2+^ oscillations. Crucially, the adjustable nature of the nsPEF parameter allows for precise control over activation signaling, a feature that chemical activators lack, as they tend to elevate Ca^2+^concentration indiscriminately and carry potential cytotoxicity. These evidences establish nsPEF as a superior AOA strategy, overcoming the limitations of existing chemical methods that fail to replicate the Ca^2+^ signaling patterns observed during fertilization.

During mammalian fertilization, the entry of sperm into the oocyte triggers a series of oscillatory Ca^2+^ changes that continue until the formation of male and female pronuclei.^41^, ^42^ Cytoplasmic Ca^2+^ dynamics exhibit hour‐scale oscillations during natural fertilization. To replicate this biological process, a prolonged Ca^2+^ oscillation profile resembling that of the IVF group was induced through repetitive nsPEF stimulation. Our results demonstrate that sustained Ca^2+^ oscillations over 2 h—elicited by either low‐ or medium‐intensity pulse protocols—significantly enhance both parthenogenetic activation efficiency and subsequent embryonic developmental competence compared to single‐pulse strategies. These findings underscore the cumulative effects of oscillatory Ca^2+^ signaling on embryogenesis and reinforce the view proposed by Ozil et al. that repetitive Ca^2+^ rises promote the gene expression involved in development and implantation.^43^, ^44^ Despite certain discrepancies between the characteristics of electrically induced [Ca^2+^]_i_ oscillations and physiological conditions, these differences did not affect the rates of oocyte activation and development, indicating that oocytes can accommodate a range of Ca^2+^ oscillatory parameters. Both transient and oscillatory Ca^2+^ signals can be decoded by oocytes to execute a variety of activation‐associated biological processes.^45^ The sustained Ca^2+^ oscillations represent an evolutionarily conserved mechanism in mammalian reproduction. Specifically, adequate Ca^2+^ signaling activates calmodulin‐dependent protein kinase II, which triggers anaphase‐promoting complex‐mediated cyclin B degradation,^14^ driving the exit from metaphase II (MII) through maturation‐promoting factor inactivation. This adaptability aligns with our findings, suggesting that oocyte activation depends on the cumulative integration of Ca^2+^ signals rather than strict signaling fidelity.^46^, ^47^ Although the role of oscillatory Ca^2+^ signals in early embryonic development remains unclear, Ozil et al. demonstrated that abnormal Ca^2+^ signals at fertilization affect gene expression in blastocysts and decrease the rate of preimplantation.^43^ Importantly, the nsPEF technology effectively addresses the dual challenges of signal accuracy and biosafety in chemical‐based approaches. Its precision in replicating sperm‐like Ca^2+^ signaling positions it as the optimal AOA strategy.

To investigate the internal mechanisms behind the [Ca^2+^]_i_ oscillations induced by nsPEF stimulation, we systematically excluded potential cytoplasmic Ca^2+^ sources on a site‐by‐site basis. Our findings revealed that the nsPEF‐induced Ca^2+^ oscillations share fundamental regulatory pathways with the physiological Ca^2+^ signaling triggered by sperm. The low‐intensity nsPEF exclusively mobilized extracellular Ca^2+^ influx, as demonstrated by the complete abolition of signals in a Ca^2+^‐free medium. This phenomenon has also been observed in studies using nsPEF pulses at relatively low intensity in other cell types.^33^, ^48^ In contrast, the moderate‐intensity nsPEF activated dual Ca^2+^ contributions: extracellular Ca influx (blocked by EGTA) and ER release (inhibited by TG). Subsequently, the Ca^2+^ levels quickly returned to baseline through backflow into the ER and mitochondria. Oocytes pretreated with Ru360 were unable to pump Ca^2+^ into the mitochondria, disrupting the original intracellular calcium regulatory homeostasis and leading to the premature interruption of the nsPEF‐induced [Ca^2+^]_i_ oscillations. Similarly, the inhibition of SOCE delayed ER refilling, prolonging the formation of spontaneous [Ca^2+^]_i_ oscillations. This confirms that both ERs and mitochondria are involved in cytoplasmic Ca^2+^ recycling and secondary replenishment during [Ca^2+^]_i_ oscillations.

Central to the maintenance of Ca^2+^ oscillations is the PIP_2_–IP_3_–IP_3_R axis, which is activated by nsPEF. The downregulation of PIP_2_ and subsequent production of IP_3_ in oocytes is caused by medium‐intensity nsPEF stimulation, leading to IP₃R‐mediated Ca^2+^ release. Inhibition of IP₃R activity leads to an early cessation of sustained Ca^2+^ oscillations by disrupting the CICR mechanism. The biphasic dependence of IP_3_R (on both Ca^2+^ levels and the production of IP_3_) gives rise to [Ca^2+^]_i_ oscillations, characterized by rapid positive feedback and slow negative feedback processes on cytosolic Ca^2+^ levels.^36^ It is plausible that PIP_2_ hydrolysis and IP_3_ generation trigger periodic Ca^2+^ release from the ERs, which explains why oocytes can still exhibit [Ca^2+^]_i_ oscillations in the absence of external Ca^2+^ (Figure 4d) or SOCE activity (Figure 4h). Additionally, the simulation model of nsPEF‐induced Ca^2+^ signaling provides a detailed explanation of how Ca^2+^ increase significantly activates IP_3_R activity at moderate intensity pulses.^36^ These mechanisms align with natural fertilization, where PLCζ‐generated IP₃ activates IP₃Rs to initiate CICR.^41^ The generation of IP_3_ through PLC‐mediated PIP_2_ hydrolysis is crucial for Ca^2+^ oscillations during fertilization across various species.^37^, ^49^, ^50^ Interestingly, transient nanopores are formed in both plasma and ER membranes upon nsPEF stimulation, allowing for the circulation of Ca^2+^ without causing irreversible damage.^33^, ^51^ This was evidenced by the restoration of membrane fluidity within 10 min after nsPEF pulses (Figure 4k). This transient permeabilization, coupled with endogenous Ca^2+^ buffering systems, enabled nsPEF to effectively leverage existing intracellular calcium homeostasis mechanisms and generate fertilization‐like Ca^2+^ signaling. These results position nsPEF as a physiologically faithful AOA strategy mitigating the diverse risks of chemical activation.

While both chemical ionophores and conventional electrical stimulation (typically employing DC pulses in 50–100 μs) have been widely used for AOA, these approaches raise concerns regarding their safety and physiological relevance.^12^, ^52^ Existing studies in this field have largely focused on feasibility and initial activation rates,^26^, ^40^, ^53^ yet few have systematically examined the underlying Ca^2+^ signaling dynamics or comprehensively assessed the technical and procedural damage inflicted on oocytes and embryos. Both ionophores and microsecond PEFs elicit only a single, transient Ca^2+^ peak,^54^ which fails to engage the intracellular signaling cascades—such as the PIP_2_–IP_3_–IP_3_R pathway—necessary for sustained Ca^2+^ oscillations. This non‐physiological Ca^2+^ response may contribute to the considerable variability in AOA outcomes reported across embryology laboratories.^13^, ^54^ More critically, such uncontrolled Ca^2+^ overload is known to disrupt cellular Ca^2+^ homeostasis, potentially leading to oxidative stress, mitochondrial dysfunction, and even the activation of Ca^2+^‐dependent apoptotic pathways.^55^, ^56^ In contrast, nsPEF activation maintains a stable cytoplasmic environment by harnessing inherent Ca^2+^ regulatory mechanisms, thereby preserving redox balance and preventing apoptosis. Mechanistic studies suggest that the capability of nsPEF to modulate transmembrane potential likely has a direct impact on the mitochondrial respiratory chain, thereby enhancing mitochondrial function and regulating ATP production.^57^, ^58^ This capacity to precisely target subcellular structures without compromising plasma membrane integrity represents a significant biosafety advantage in AOA.

Notably, nsPEF elicits dose‐dependent Ca^2+^ transients in human unfertilized oocytes, underscoring its broad potential applications. The single‐pulse nsPEF generates Ca^2+^ spikes that exhibit a linear correlation with both field intensity and pulse number. Repeated nsPEF stimulation induces Ca^2+^ oscillations in human oocytes (Figure 7c). The vitrified IVM oocytes demonstrated attenuated Ca^2+^ responses, failing to replicate sustained Ca^2+^ oscillations, which can be attributed to suboptimal cytoplasmic conditions and inadequate Ca^2+^ stores.^59^ However, it is anticipated that applying specific quantities of nsPEF pulses at precise intervals will accurately replicate [Ca^2+^]_i_ oscillations during human fertilization. The observed disparities in nsPEF intensity between human and mouse oocytes may be due to variations in cell diameters, as the penetration ability of nsPEF into spherical cells follows the cell diameter–electric field relationship.^60^ Future studies should focus on optimizing nsPEF parameters to accommodate interspecific ER heterogeneity and intracellular Ca^2+^ storage capacity.

While calcium ionophore A23187 remains a mainstream method for clinical AOA,^16^, ^17^ significant limitations persist regarding its induction of non‐physiological Ca^2+^ elevation and oxidative cytotoxicity.^61^ The nsPEF activation offers a unique combination of precision and biosafety and presents an unprecedented solution to overcome OAD, particularly in cases of severe male factor infertility or TFF. The capability of nsPEF to enable quantifiable and programmable control of intracellular Ca^2+^ signaling is unmatched by existing AOA methods, which mitigate safety concerns associated with current techniques and maximize the developmental potential. Beyond immediate clinical applications, nsPEF serves as a promising tool for investigating activation mechanisms across different patient profiles (e.g., PCOS patients), facilitating future development of personalized activation protocols. We acknowledge that current nsPEF systems involve considerable operational complexity. To transition this technology toward clinical adoption, efforts should focus on developing user‐friendly medical‐grade nsPEF systems, conducting rigorous clinical trials to validate live birth outcomes, and implementing long‐term follow‐up studies. With these advancements, the nsPEF‐based activation will become an efficient and safe strategy that may redefine the technical standards for AOA.

Additionally, the development of nsPEF technology could revolutionize embryogenesis techniques, including somatic cell nuclear transfer (SCNT)^62^, ^63^ and germ cell differentiation,^64^, ^65^ processes involving Ca^2+^‐dependent regulatory networks.^46^, ^62^, ^63^ Specifically, nsPEF could offer a novel and precise artificial activation method in SCNT protocols, significantly improving reprogramming efficiency.^66^, ^67^ The unique Ca^2+^ dynamics required for germ cell proliferation and differentiation may be faithfully replicated using nsPEF‐based stimulation strategies. Evidence from prior studies supports this supposition—for instance, nsPEF‐triggered ion fluxes have been shown to guide lineage‐specific differentiation in mesenchymal stem cells.^68^, ^69^ Furthermore, the ability of nsPEF to manage Ca^2+^ dynamics may also extend to the field of neural signaling, providing new insights into the mechanisms of calcium dysregulation associated with neurodegenerative diseases. As nsPEF technology evolves, collaborative efforts across various disciplines will unlock its full potential, bridging gaps among reproductive medicine, neurology, and developmental biology.

## 4 CONCLUSION

In summary, we systematically investigated the relationship between [Ca^2+^]_i_ signaling profile, which is finely modulated by the nsPEF intensity, and oocyte activation and development. Moderate‐intensity nsPEF effectively mobilizes intracellular calcium stores, generating a distinct pattern of cytoplasmic Ca^2+^ oscillations in a physiological manner. This process promotes the completion of meiosis and early embryonic development. Additionally, we have identified the electrical intensity threshold of nsPEF stimulation that regulates Ca^2+^ changes in human oocytes. These findings position nsPEF as a promising alternative to chemical activators, offering high safety and efficacy while minimizing off‐target effects, thereby enhancing the clinical potential of AOA technologies.

## 5 METHODS

*Study design*: The objective of this study was to establish the effects of cytoplasmic Ca^2+^ response modulated by nsPEF stimulation on assisted oocyte activation and embryo development. Mature oocytes were collected from the murine oviducts and randomly allocated into distinct experimental cohorts, including nsPEF‐treated groups subjected to stimulation of varying intensities and A23187‐treated oocytes as positive controls. Real‐time monitoring of cytoplasmic Ca^2+^ concentration changes via confocal microscopy for 2 h during nsPEF stimulation, with subsequent analysis of the elicited Ca^2+^ oscillation patterns. Additionally, calcium imaging data were collected on untreated oocytes and those treated with specific inhibitors to identify the origins of Ca^2+^ oscillation formation. The pathways PIP_2_–IP_3_–IP_3_R that maintain Ca^2+^ oscillations were explored using a GFP‐C1‐PLCδ‐PH DNA construct. Comprehensive assessment of oocyte activation post‐nsPEF exposure, including morphological examination, live‐cell staining, and immunofluorescence analysis to determine survival rates, cytoplasmic integrity, and mitochondrial function at 1‐h post‐stimulation. Furthermore, the human oocytes underwent nsPEF stimulation under optimized parameters, and the resulting Ca^2+^ oscillation patterns were analyzed using time‐lapse Ca^2+^ imaging to corroborate the findings established in the murine model.

*Ethics Statement*: This study was registered at the Medical Ethics Committee of Peking University People's Hospital (PKUPH) prior to inclusion (No. 2020PHB397‐01), and written informed consent was obtained from patients undergoing controlled ovarian hyperstimulation for ICSI treatment. All procedures involving live animal handling and euthanasia were approved by the Institutional Animal Care and Use Committee and Ethic Review Committee of PKUPH (No. 2020PHE099) and the Ethic Review Committee of Beijing Chao‐Yang Hospital, Capital Medical University (No. 2022‐KE‐436). Unless otherwise stated, all chemicals and medicines were purchased from the Sigma Chemical Co. (St Louis).

*Animal experiments*: Seven‐week‐old CD‐1® (ICR) mice were acquired from the Beijing Vital River Laboratory Animal Co., Ltd., and housed under standard conditions in the animal experimentation facility. These mice were maintained in a pathogen‐free environment with 12‐h light/dark cycles for 2 weeks. The mice were marked by ear tagging and randomly assigned to experimental groups using computer‐generated randomization before the experiment. A total of 200 female ICR mice were used as oocyte donors for this study. Mice were randomly assigned to experimental groups. From these animals, approximately 2500 MII oocytes were collected and subjected to the various treatments described.

*Mouse oocyte collection and culture in vitro*: Female 9‐week‐old mice that were healthy, active, and exhibited a slightly opened pink vaginal orifice were selected. To minimize animal suffering, mice were euthanized by cervical dislocation in accordance with accepted animal welfare guidelines. Mouse mature oocytes were harvested following ovulation induction (*n* = 9 per group), which involved the intraperitoneal injection of 10 IU of pregnant mare serum gonadotropin (PMSG), followed 46–48 h later by an injection of 10 IU of human chorionic gonadotropin (hCG), both from Ningbo Second Hormone Factory. The oocytes in MII stage were released from the oviducts at 14‐h post‐hCG administration and cumulus cells were dissociated using 0.1% (w/v) hyaluronidase. The oocyte exhibiting abnormal morphology or size was excluded. Only oocytes that had extruded the first polar body were selected and randomly allocated into several groups. All treatments for each group were completed within 30 min. This entire procedure was considered one biological replicate, and a minimum of three biological replicates was performed per experiment. The cumulus‐free oocytes were then washed three times and maintained in M2 medium under mineral oil at 37°C and 5% CO_2_ until further experimental procedures. The sample size for each experiment was determined a priori using power analysis to ensure sufficient statistical power. Power calculations were performed based on effect sizes observed in our preliminary data. We utilized G*Power software to determine the minimum sample size required. Effect sizes (d) were selected according to conventions established by J. Cohen in *Statistical Power Analysis for the Behavioral Sciences*.^70^ For comparisons between two groups using a *t*‐test, we aimed to detect a large effect size (*d* = 1.0) with a statistical power (1 − *β*) of 0.8 and a significance level (*α*) of 0.05. This analysis indicated a minimum requirement of 14 cells per group. For comparisons across multiple groups using ANOVA (*F*‐test), a target effect size of *f* = 0.4 (large), with the same power and alpha thresholds, was applied. The analysis determined that 16–19 oocytes per group were required.

*Nanosecond pulse exposure on mouse oocytes*: Oocytes were collected and randomly grouped, then they were stimulated by low‐, medium‐, or high‐intensity nsPEF. A custom‐built nsPEF generator, delivering pulses of 10 ns duration, was connected to a petri dish with parallel electrodes to apply pulses on the oocytes (Figure S2A,B). Oocytes were subjected to 10 pulses of nsPEF at different intensities, each with a duration of 10 ns. The intervals between pulses were approximately 1 s.

Considering the different volumes of the M16 medium used in the self‐made electrode dish (Figure S2C) versus the platinum Petri dish (Harvard Bioscience, MA), we established a relationship between the electric pulses in different electrode dishes based on the electrical doses of nsPEF. The electrical dose of nsPEF is chiefly determined by pulse intensity, field duration, and pulse count, while the resistance of the medium between parallel electrodes also influences the electrical dose exposed by oocytes. The formula for calculating the electrical dose between parallel electrodes is as follows:

(1) $$\mathrm{AD}=\frac{E^{2}\times d^{2}\times W\times n}{M\times R}=U^{2}\times n\times \frac{W}{M\times R}.$$

Here, AD represents the absorbed dose (J/g), which indicates the electrical dose from nsPEF stimuli; *E* is the applied nsPEF's pulse intensity (V/m); *d* stands for the pulse electrode gap (m); *W* signifies the pulse width (s); *n* is the number of nsPEF pulses applied; *R* depicts the resistance of the culture medium, and M represents the volume of solution between the parallel electrodes. Consequently, the relationship between the AD in different stimulation dishes is exhibited in Table S1.

Specifically, the low‐intensity group manifested the electric dose of 7–8 kV/cm applied to the platinum dish was equivalent to the electric dose of 16–17 kV/cm in the self‐made confocal dish; the medium‐intensity group manifested the equal electric dose of 9.5–10.5 kV/cm in the platinum dish and 20–21 kV/cm in the self‐made confocal dish; the high‐intensity group manifested the equal electric dose of 12.5–14 kV/cm in the platinum dish and 24–25 kV/cm in the self‐made confocal dish.

*Mouse oocyte activation and in vitro culture*: Following exposure to different nsPEF pulses, oocytes were thoroughly washed and cultured in KSOM medium under mineral oil at 37°C in a humidified atmosphere containing 5% CO_2_ during embryonic development. Oocytes were incubated in M16 medium containing 10 μM A23187 for 10 min at 37°C and 5% CO_2_ as a positive control, and in vitro culture in the same protocol. Oocytes placed in Petri dishes but exposed to 0 kV/cm pulses served as the sham exposure group (control group). Oocytes that form 1–2 pronuclei or cleave were considered as activated oocytes, and they were cultured in KSOM medium and tracked at 24‐h intervals for up to 5.5 days to monitor embryo development. Each treatment group contained 15–20 oocytes per replication. The data presented were derived from five independent replicate experiments. In total, approximately 600 oocytes collected from 30 female mice were used in this study.

*Measurement of intracellular ROS, GSH, and oocyte apoptosis*: Intracellular ROS or GSH levels in oocytes were detected using 1 mmol/L 2′,7′‐dichlorodihydrofluorescein diacetate or 10 μmol/L Cell Tracker Blue (Invitrogen, Carlsbad, CA). Images were captured using a fluorescence microscope (Olympus IX73) and quantified with EZ‐C1 Free‐Viewer software (Nikon, Tokyo, Japan). To assess early apoptosis in oocytes, staining of oocytes was performed with the Annexin V‐FITC/PI Apoptosis Detection Kit (Vazyme, Nanjing, China), following the manufacturer's protocol. Oocytes were incubated with 5 μL of Annexin V‐FITC (Fluorescein Isothiocyanate) and 5 μL of propidium iodide (PI) for 10 min in the dark at 37°C, then thoroughly washed three times with PBS (Phosphate Buffered Saline). Green fluorescence localized to the zona pellucida indicated non‐apoptotic cells, while green fluorescence present on both the membrane and zona pellucida signaled apoptosis. PI can penetrate the cell membrane of necrotic or late apoptotic cells, staining the nucleus with red fluorescence. The fluorescence signals were visualized using a fluorescence microscope (Olympus IX73). Approximately 20 oocytes from three different batches were used in each treatment group.

*Measurement of MMP and ATP level*: Oocytes were incubated with 10 μM Image‐iT™ tetramethylrhodamine (Invitrogen, Belgium) in M16 medium at 37°C for 30 min to measure the MMP level in oocytes. Subsequently, the stained oocytes were washed and examined using a fluorescence microscope (Olympus IX73). Approximately, 20 oocytes from three different batches were used in each treatment group. ATP content within individual oocytes was quantified using an Enhanced ATP Assay Kit (Beyotime Institute of Biotechnology, Shanghai, China), following the manufacturer's protocol. A series of ATP standards was prepared, encompassing a range from 0 to 40 pmol ATP. Oocytes were lysed by treatment with 20 μM lysis buffer in 0.2‐mL RNA‐free centrifuge tubes, followed by centrifugation at 4°C and 12,000*g* for 5 min. Unless specified, all procedures were performed on ice. ATP detection solution was dispensed into 96‐well plates and incubated at room temperature for 3–5 min. In each well, standard solutions and ATP detection diluents were introduced. Samples were subsequently added to the wells, and luminescence was promptly measured using a luminometer (Infinite F200, Tecan, Switzerland). ATP concentrations were determined from the standard curves (Figure S3). The total ATP level was divided by the count of oocytes in each sample to derive the average ATP content per oocyte (pmol). Denuded oocytes were incubated in M16 medium containing CCCP (Beyotime, China) for 2 h, followed by determination of MMP level or ATP content, as the positive control group.

*Immunofluorescence*: Oocytes/embryos were fixed in 4% (w/v) paraformaldehyde for a minimum of 24 h at 4°C, followed by permeabilization with 0.5% Triton X‐100 in PBS–0.1% PVA at room temperature for 1 h and washed three times with washing buffer (PBS–0.1% PVA containing 0.1% Triton X‐100). Oocytes/embryos were subsequently incubated in blocking buffer (3% BSA in washing buffer) for 1 h at room temperature and incubated at 4°C overnight with different primary antibodies (anti‐CDX2, 1:500, for TE; anti‐Nanog, 1:1000 for ICM; anti‐γH2A.X, 1:100, for DNA double‐strand break, Abcam, Cambridge, England) diluted in blocking buffer. After being thoroughly washed, the oocytes/embryos were further incubated with the appropriate secondary antibody (goat anti‐mouse FITC‐conjugated antibody, 1:100, goat anti‐rabbit Alexa Fluor™ 594‐conjugated antibody, 1:100, Abcam, England) for 1 h at 37°C, then washed four times in washing buffer. DNA was subsequently stained with 4′,6‐diamidino‐2‐phenylindole (Vector Laboratories Inc., Burlingame, CA). The stained oocytes/embryos were mounted on glass slides and observed using laser‐scanning confocal microscopy (Nikon A1R), with qualitative analysis performed using NIS‐Elements AR software (Nikon Instruments).

Oocytes were incubated with 20 mg/mL zeocin in M16 medium at 37°C for 30 min to induce DNA damage. Oocytes exhibiting the anti‐γH2A.X signal under confocal microscopy were considered as positive cells for DNA damage. The percentage of positive cells and the fluorescence intensity of these positive cells were calculated separately for each group to assess the degree of DNA damage.

*Measuring cytosolic Ca* ^*2+*^ *and mitochondrial Ca* ^*2+*^ *alteration*: Cytosolic Ca^2+^ levels were assessed using the cytoplasmic Ca^2+^ probe Fluo‐4 AM (Cat. No. 40704ES50, Yeasen, Shanghai, China). The oocytes were processed in M2 medium with 5 μM Flou‐4 AM for 20 min and washed three times with DPBS. Subsequently, they were placed within a self‐made 1‐mm‐gap electrode confocal dish (Figure S2C) and stimulated as previously explained on the stage of a confocal laser scanning microscope (Nikon A1R). Similarly, to assess the dynamics of mitochondrial Ca^2+^ concentration, oocytes were incubated with 5 μM Rhod‐2 AM (Cat. No. R1244, Invitrogen) for 30 min at 37°C. Fluo‐4 AM or Rhod‐2 AM was excited using wavelengths of 488 or 552 nm, and the emitted fluorescence was collected at wavelengths of 520 or 580 nm.

Images were acquired at 4‐s intervals over approximately 30 min, and analyzed intracellular fluorescence intensity by ImageJ software. The fluorescence ratio *F*/*F*_0_, defined as the fluorescence intensity (*F*) of the Ca^2+^ indicator at a specific timepoint during the measurement normalized to the baseline intensity before exposure (*F*_0_), served to illustrate the dynamics of [Ca^2+^]_i_ or intra‐mitochondrial Ca^2+^ ([Ca^2+^]_mito_). In all experiments, nsPEF exposure was initiated following a 1‐min delay to establish a baseline fluorescence level.

*Specific inhibitor treatment*: The oocytes were incubated in M16 medium with 5 mM EGTA (Yeasen, China) or 10 μM Thapsigargin (TG, MedChemExpress, Shanghai, China) at 37°C for 30 min to deplete Ca^2+^ in the extracellular medium or ER store separately. XC (MedChemExpress, China) is a selective inhibitor of the IP3R on ERs. Oocytes were placed in M16 medium with 10 μM XC for 60 min to inhibit IP3R activity. The effect of SOCE in oocytes was inhibited using 2‐aminoethyl diphenylborinate (2‐APB, MedChemExpress, China). The oocytes were incubated with 10 μM 2‐APB for 30 min in M16 medium.

*In vitro fertilization*: Sperm for IVF procedures were obtained from 9‐week‐old male CD1 mice. The cauda epididymis of the sacrificed was collected and separated with scissors in 100 μL Human Tubal Fluid (HTF) medium. The sperm released from the epididymis were incubated for 1 h at 37°C and 5% CO_2_ conditions. Cumulus‐free oocytes were transferred to 90 μL drops of HTF medium, followed by the addition of 1 × 10^5^ sperm/mL. After a 15‐min incubation, excess sperm were washed away, and oocytes were either loaded with Fluo‐4 AM for [Ca^2+^]_i_ monitoring or cultured in vitro for embryo development as previously described.

*Plasmid microinjection*: Oocytes were transfected with the GFP‐C1‐PLCδ‐PH DNA construct, which consists of the Pleckstrin homology (PH) domain of PLCδ (with tagged IP_3_ or PIP_2_) fused with a green fluorescent protein (GFP) (Addgene plasmid 21179). The microinjection of an optimal plasmid concentration (150 μM) was performed on germinal vesicle (GV) stage oocytes using a microinjection instrument (Eppendorf, Hamburg), followed by a 30‐min incubation period. Subsequently, the oocytes were arrested at the GV stage in M16 medium supplemented with 2.5 μM milrinone for 4 h. Following thorough washing, the oocytes were cultured in M16 medium under mineral oil at 37°C and incubated in a 5% CO_2_ atmosphere during the GV to MII stages for 12–14 h. Finally, MII oocytes with GFP‐C1‐PLCδ‐PH signals were placed in a self‐made electrode dish and exposed to 10 ns pulses with medium intensity. The laser scanning fluorescence images were acquired at a rate of 1 image every 4 s for 30 min.

*Human oocyte collection*: Immature oocytes from patients undergoing ICSI/IVF treatment at the Beijing Chao‐Yang Hospital were used in the study. A total of 9 patients (oocyte donors) underwent controlled ovarian stimulation (COS) using the gonadotropin‐releasing hormone (GnRH) antagonist protocol. The COS was initiated with a dose of gonadotropins (Gonal‐F, Merck, Germany; 150–300 IU/day) on day 2–3 of the menstrual cycle. Gn dosages were adjusted according to the ovarian responses evaluated by transvaginal ultrasonography and total serum levels of estradiol (E2) and luteinizing hormone (LH). A GnRH antagonist (Cetrotide, Merck, Germany; 0.25 mg/day) was provided when the leading follicle diameter was 13–14 mm. Once at least two follicle diameters reached 18 mm, triptorelin acetate (Decapeptyl, Ferring, Switzerland, 0.2 mg) and hCG (Merck, Germany; 6500 IU) were used to trigger follicle maturation. Cumulus‐Oocyte Complexes (COCs) were retrieved at 36 h later with ultrasound‐guided transvaginal follicular aspiration.

*Human oocyte in vitro maturation*: The immature oocytes of GV oocytes and meiosis I stage oocytes were cultured in 20 μL droplets of G‐2Plus medium (Vitrolife, Sweden) supplemented with 0.1 IU/mL FSH (Puregon) under paraffin oil. After 24–48 h cultured at 37°C, 6% CO_2_, 5% O_2_, and 89% N_2_, IVM–MII oocytes were frozen using Cryotop methodology with vitrification kits (Kitazato Corporation, Japan).

*NsPEF exposure on human oocytes and calcium imaging measurement*: Human thawed oocytes were incubated at 37°C for 30 min in G‐1Plus medium supplemented with 5 μM Fluo‐4 AM. Subsequently, the oocytes were transferred to G‐1Plus medium between electrodes and were subjected to nsPEF pulses with different parameters. Due to the different sizes and states of human and mouse oocytes, we stimulated human oocytes with nsPEF at low (20 kV/cm), medium (23 kV/cm) and high (25 kV/cm) intensity with 10–50 pulses, respectively. The intervals between pulses were approximately 1 s.

*Statistical analysis*: All experiments were repeated at least three times. Data are presented as means ± *SEM*, unless otherwise stated. Statistical analyses were performed using SPSS 20. The normality of all data sets was assessed using the Shapiro–Wilk test. Data that did not follow a normal distribution were analyzed using non‐parametric tests (Kruskal–Wallis test followed by Dunn's post hoc test), and the normally distributed data were subjected to Student's *t*‐test or one‐way ANOVA. The homogeneity of variances was verified, and either the ordinary ANOVA test (when variances were homogeneous, *p* > 0.05) or the Brown–Forsythe and Welch ANOVA tests (when variances were unequal, *p* < 0.05) were applied accordingly. The *p* < 0.05 was considered statistically significant.

## AUTHOR CONTRIBUTIONS

Y.‐D.S. participated in study design, execution, analysis, and manuscript drafting. T.A. and R.L. participated in patient oocyte collection, in vitro maturation, and culture, and revised the manuscript. Y.‐W.L., H.‐Z.X., Y.‐X.Z., and X.‐W.F. assisted with data analysis and revised the manuscript. L.F. participated in patient oocyte collection. S.H., Z.‐Y.‐S., X.‐Y.B., and Y.F. assisted with patient oocyte collection. Y.‐P.H. and Q.L. conceived the idea, planned the study, and critically revised the manuscript. All authors read and agreed to the published version of the article.

## FUNDING INFORMATION

This study was supported by the National Natural Science Foundation of China (Grant No. 82071715).

## CONFLICT OF INTEREST STATEMENT

The authors declare no conflicts of interest.

## Supporting information


**Figure S1:** Mitochondria are involved in nsPEF‐induced sustained [Ca^2+^]_i_ oscillations.
**Figure S2:** The utilization of the nsPEF generator for the oocyte.
**Figure S3:** The standard curve with known concentrations of ATP.
**Table S1:** The electrical dose in different dishes by nsPEF stimulation.
**Material S1:** The description of the process flow of a self‐made dish.

**Video S1:** Sustained cytoplasmic Ca^2+^ oscillations induced by nsPEF stimulation at medium intensity, related to Figure 1.

## Data Availability

The data that support the findings of this study are available from the corresponding author upon reasonable request.
