# Supplementary material for: Precise mimicry of physiological Ca2+ oscillations for mammalian oocyte activation by nanosecond pulsed electric field
Source: Bioeng Transl Med. 2025 Dec 5;11(1):e70094. doi: 10.1002/btm2.70094 (PMC12821223; doi:10.1002/btm2.70094)
Supplement: Supplementary file 1 — Figure S1: Mitochondria are involved in nsPEF‐induced sustained [Ca2+]i oscillations. Figure S2: The utilization of the nsPEF generator for the oocyte. Figure S3: The standard curve with known concentrations of ATP. Table S1: The electrical dose in different dishes by nsPEF stimulation. Material S1: The description of the process flow of a self‐made dish. [file BTM2-11-e70094-s002.docx]

Supporting Information


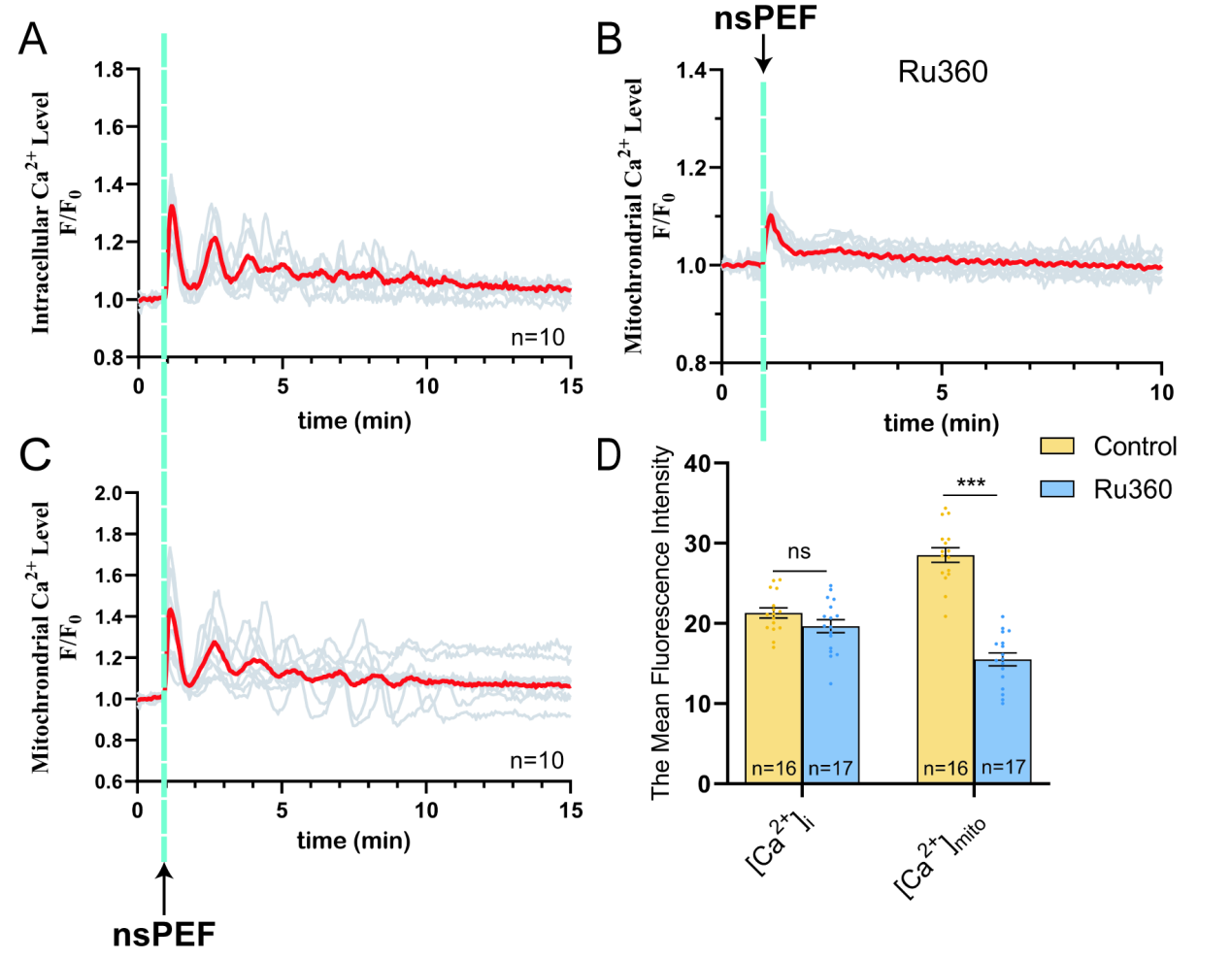


Figure S1. Mitochondria are involved in nsPEFs-induced sustained [Ca^2+^]_i_ oscillations

(A-C) The patterns of cytoplasmic Ca^2+^ dynamics (A) and mitochodrial Ca^2+^ dynamics in mouse oocytes with and without 5 μM Ru360 (B-C) after medium-intensity nsPEF exposure. (D) Quantification of the relative levels of cytoplasmic Ca^2+^ and mitochondrial Ca^2+^ in oocytes oocytes incubated with 5 μM Ru360. ****P* < 0.001; ns indicates non-significant (*P* > 0.05). Error bars show SEM.


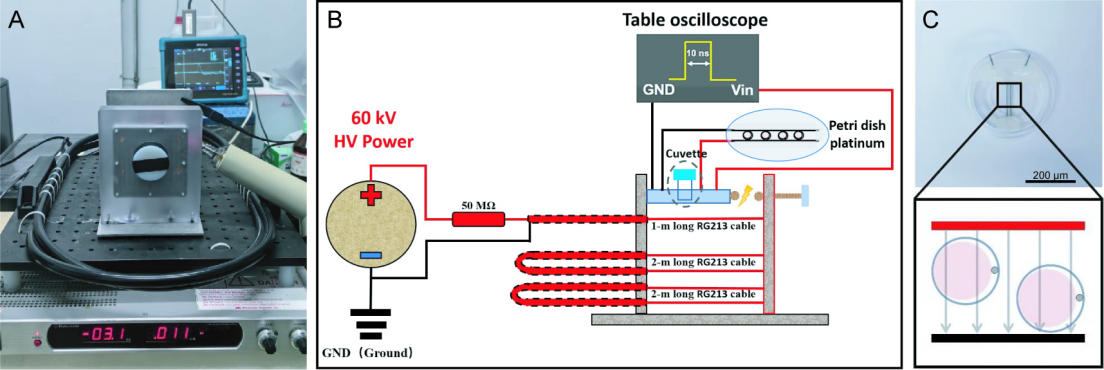


Figure S2. The utilization of the nsPEF generator for the oocyte

(A) The represent figure of custom-built nanosecond pulsed electric field (nsPEF) generator (B) The schematic diagram of nsPEF. Two cable lengths of 2 m and one cable length of 1 m are chosen in order to generate 10-ns pulses. HV: high voltage, GND: ground, Vin: input voltage. (C) The diagram of the self-made confocal dish equipped with electrodes allowing for both nsPEF pulse generation and real-time Ca^2+^ imaging. The figure (below) shows the position of the microelectrode relative to the oocytes location. Oocytes are subjected to the uniform electric field between parallel electrodes. Scale Bar: 200 μm


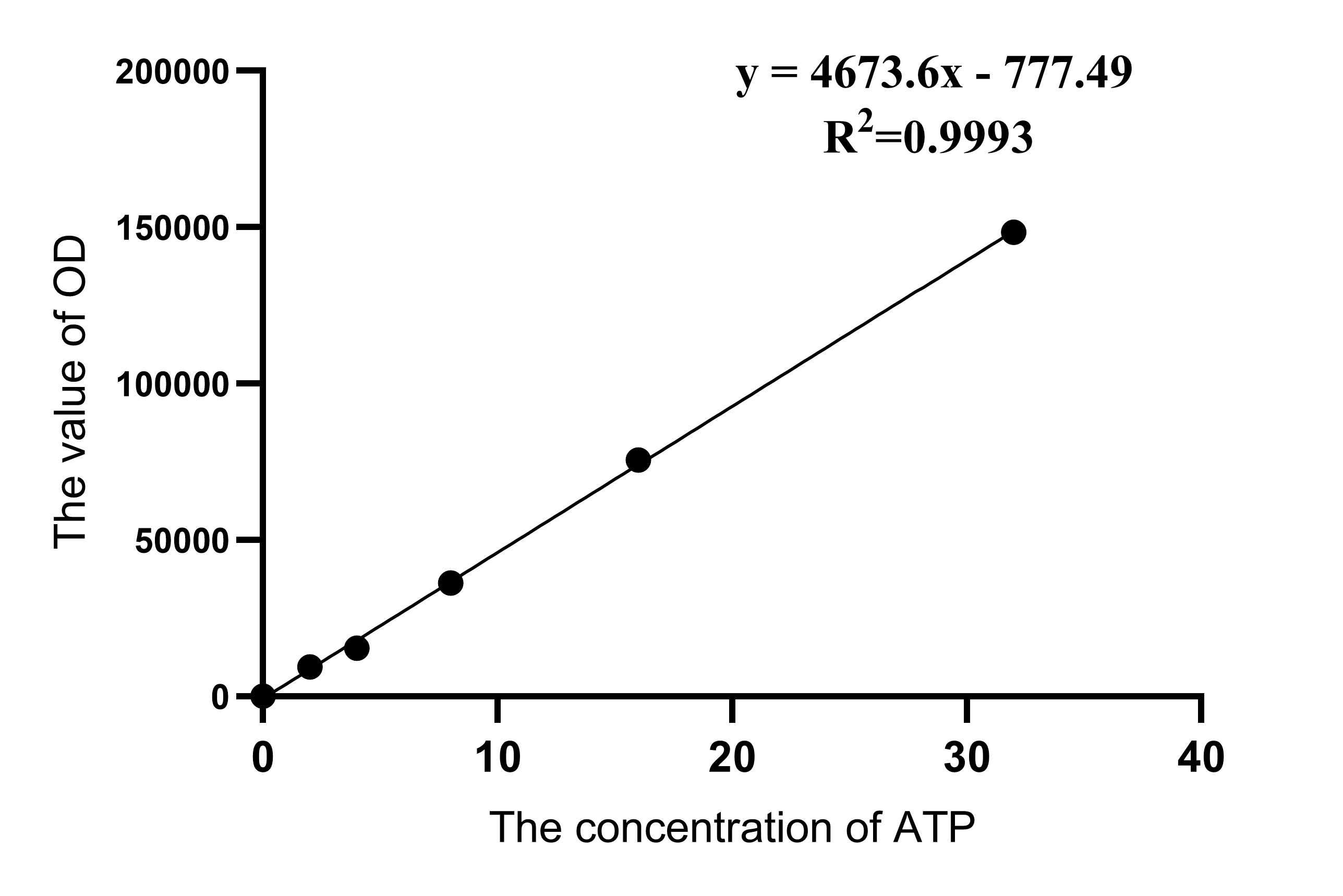


Figure S3. The standard curve with known concentrations of ATP

Table S1. The electrical dose in different dishes by nsPEF stimulation

|  | nsPEF stimulation at low intensity | nsPEF stimulation at medium intensity | nsPEF stimulation at high intensity |
| --- | --- | --- | --- |
| Pulse Intensity in the Platinum Petri dish | 7-8 kV/cm | 9.5-10.5 kV/cm | 12.5-14 kV/cm |
| Pulse Intensity in the self-made electrode confocol dish | 16-17 kV/cm | 20-21 kV/cm | 24-25 kV/cm |
| AD (×W/R, J/g) | ~2.0×10^6^ | ~3.2×10^6^ | ~4.5×10^6^ |

Material S1. The description of the process flow of a self-made dish

The self-made electrode dish (Figure S2C) was designed to address the limitations of commercial systems. It consisted of two parallel electrodes with a 1 mm gap, fabricated on a 15 mm diameter glass coverslip (thickness < 19 μm) mounted within a standard 30 mm culture dish. Both ends of the electrode pair were sealed to form a stable microchamber. The chamber was filled with culture medium and overlaid with mineral oil to prevent evaporation. After temperature equilibration, individual oocytes were carefully placed between the electrodes. nsPEF activation was then applied under confocal microscopy observation, with a specific energy dosage ranging from 2-4×10⁶ J/g.

Video S1. Sustained cytoplasmic Ca^2+^ oscillations induced by nsPEF stimulation at medium intensity
